# Comparative transcriptomic analysis of SARS-CoV-2 infected cell model systems reveals differential innate immune responses

**DOI:** 10.1038/s41598-021-96462-w

**Published:** 2021-08-25

**Authors:** Guihua Sun, Qi Cui, Gustavo Garcia, Cheng Wang, Mingzi Zhang, Vaithilingaraja Arumugaswami, Arthur D. Riggs, Yanhong Shi

**Affiliations:** 1 Department of Diabetes Complications & Metabolism, Beckman Research Institute of City of Hope, Duarte, CA 91010 USA; 2 Division of Stem Cell Biology Research, Department of Developmental and Stem Cell Biology, Beckman Research Institute of City of Hope, Duarte, CA 91010 USA; 3 Department of Molecular and Medical Pharmacology, University of California, Los Angeles, Los Angeles, CA 90095 USA; 4 Eli and Edythe Broad Center of Regenerative Medicine and Stem Cell Research, University of California, Los Angeles, CA 90095 USA

**Keywords:** Cell biology, Computational biology and bioinformatics, Immunology, Microbiology, Molecular biology, Systems biology, Diseases, Pathogenesis

## Abstract

The transcriptome of SARS-CoV-2-infected cells that reflects the interplay between host and virus has provided valuable insights into mechanisms underlying SARS-CoV-2 infection and COVID-19 disease progression. In this study, we show that SARS-CoV-2 can establish a robust infection in HEK293T cells that overexpress human angiotensin-converting enzyme 2 (hACE2) without triggering significant host immune response. Instead, endoplasmic reticulum stress and unfolded protein response-related pathways are predominantly activated. By comparing our data with published transcriptome of SARS-CoV-2 infection in other cell lines, we found that the expression level of hACE2 directly correlates with the viral load in infected cells but not with the scale of immune responses. Only cells that express high level of endogenous hACE2 exhibit an extensive immune attack even with a low viral load. Therefore, the infection route may be critical for the extent of the immune response, thus the severity of COVID-19 disease status.

## Introduction

Coronavirus (CoV) disease 2019 (COVID-19) is a pandemic caused by severe acute respiratory syndrome CoV 2 (SARS-CoV-2)^1,2^. Although enormous information has been gathered about this virus for the past year, there are still many unknowns. Scientists and medical doctors are rigorously developing ways to prevent SARS-CoV-2 infection, manage disease development, and apply relevant therapeutic strategies to patients with different extent of symptoms. Despite of rapid deployment of vaccines that could prevent further spread of the virus and eventually take the pandemic under control, an effective cure is still not available. Unlike the two previous deadly CoV diseases, including SARS that was caused by SARS-CoV and middle east respiratory syndrome (MERS) that was caused by MERS-CoV, COVID-19 caused by SARS-CoV-2 infection exhibits a much wider range of disease symptoms because SARS-CoV-2 is much more contagious and can be incubated longer, but has a lower death rate.

While all three pathogenic CoVs can cause similar symptoms, including fever, cough, difficulty in breathing, SARS-CoV-2 also causes additional symptoms, such as loss of taste or smell^3–6^. The infection by SARS-CoV-2 leads to about 80% non-severe cases (40% asymptomatic and 40% mild symptoms) and 20% severe cases (15% severe illness cases and 5% cases at critical condition that results in around 2 to 3% death globally)^7–9^. In addition, SARS-CoV-2 can cause lingering or recurring post COVID-19 symptoms, the so-called long COVID, including unusual fatigue, brain fog, and insomnia that can persist for a long period of time^10–13^. In some asymptomatic or mildly affected patients and immunocompromised patients, the SARS-CoV-2 virus can persist for a long period of time and accumulate many mutations that may present a hurdle to have the pandemic under control^14–17^.

Although detailed disease mechanisms underlying the wide variety of COVID-19 symptoms are lacking, it is generally accepted that SARS-CoV-2 infection primarily damages the lungs and can also cause severe problems to heart, kidney, gastrointestinal tract, and brain. Viral infection can induce various host responses that are reflected as changes in host transcriptome, epigenome, and proteome. Identifying these changes can provide critical information at molecular level to understand the cause of symptoms and develop therapeutic strategies accordingly.

Since the beginning of the pandemic, studies that were mostly focused on severe COVID-19 cases have uncovered changes in host transcriptome, epigenome, and proteome upon SARS-CoV-2 infection and have provided strong evidence that the disease state is correlated with inadequate or defective host immune responses^18–26^. The abnormal host immune response includes overreaction of pro-inflammatory genes and lack of adequate anti-inflammatory gene expression, which leads to severe symptoms and systemic hyperinflammation in patients with severe cases. These studies revealed cytokine release syndrome, an acute systemic inflammatory syndrome, as the underlying immunopathology in most severe COVID-19 cases. Results from these omics studies are further supported by animal model studies and clinical data from patients with genetic mutations in immune genes that cannot mount effective Type I or Type III interferon responses^27–31^. However, not much resource has been applied to study symptoms in non-severe cases and the cause of post COVID-19 symptoms remains elusive. The large population of patients with non-severe COVID-19 symptoms represents a significant public health concern, therefore studies to assess these symptoms and to find ways to prevent these symptoms and their potential long-lasting effects, and to develop appropriate treatments are urgently needed.

The published transcriptome data in many cell lines and patient samples have shown that immune response genes are overwhelmingly represented in genes that are differentially expressed (DEGs) between control and SARS-CoV-2-infected cells, which makes it difficult to identify host response genes beyond immunity. In the current study, we chose HEK293T cells that are known to have innate immunity defects to identify non-immune response genes that are induced in response to SARS-CoV-2 infection^32,33^. Despite the very high viral load, SARS-CoV-2 infection in HEK293T cells that overexpress human angiotensin-converting enzyme 2 (hACE2) (HEK293T-hACE2) caused very low level of immune responses. Instead, the unfolded protein response (UPR), a cellular response of the endoplasmic reticulum (ER) stress, was highly activated. A set of gene expression changes observed in this transcriptome matched with gene expression changes in the transcriptome of SARS-CoV-2 infected individuals who do not undergo severe host immune response, such as asymptomatic individuals or COVID-19 patients with mild symptoms or post infection symptoms. Moreover, we have identified a list of host genes that are common to COVID-19 symptoms by comparing transcriptomic datasets in SARS-CoV-2-infected cells of different tissue types with or without host immune response.

## Results

### SARS-CoV-2-infected HEK293T-hACE2 cells generated a transcriptome without strong immune response

**Figure 1:**
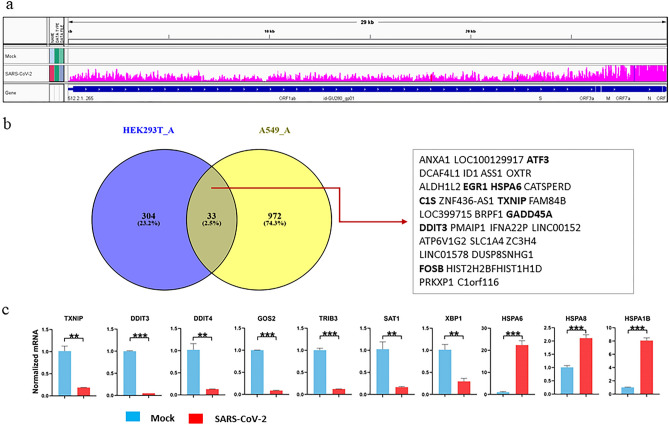
SARS-CoV-2 infection of HEK293T-hACE2 (HEK293T_A) cells and qPCR validation of DEGs. (**a**) IGV view of SARS-CoV-2 infection in HEK293_A cells: reads mapped to SARS-CoV-2 genome. (**b**) DEGs overlapped in HEK293T_A versus A549-hACE2 (A549_A) infection. (**c**) qPCR validation of DEGs in HEK293T_A infection. Statistical significance was analyzed using GraphPad Prism 8. Unpaired Student’s t test was used. For all test, p values were presented as ∗ *P* < 0.05, ∗∗ *P* < 0.01, and ∗∗∗ *P* < 0.001. Error bar stands for SD.

**Figure 2:**
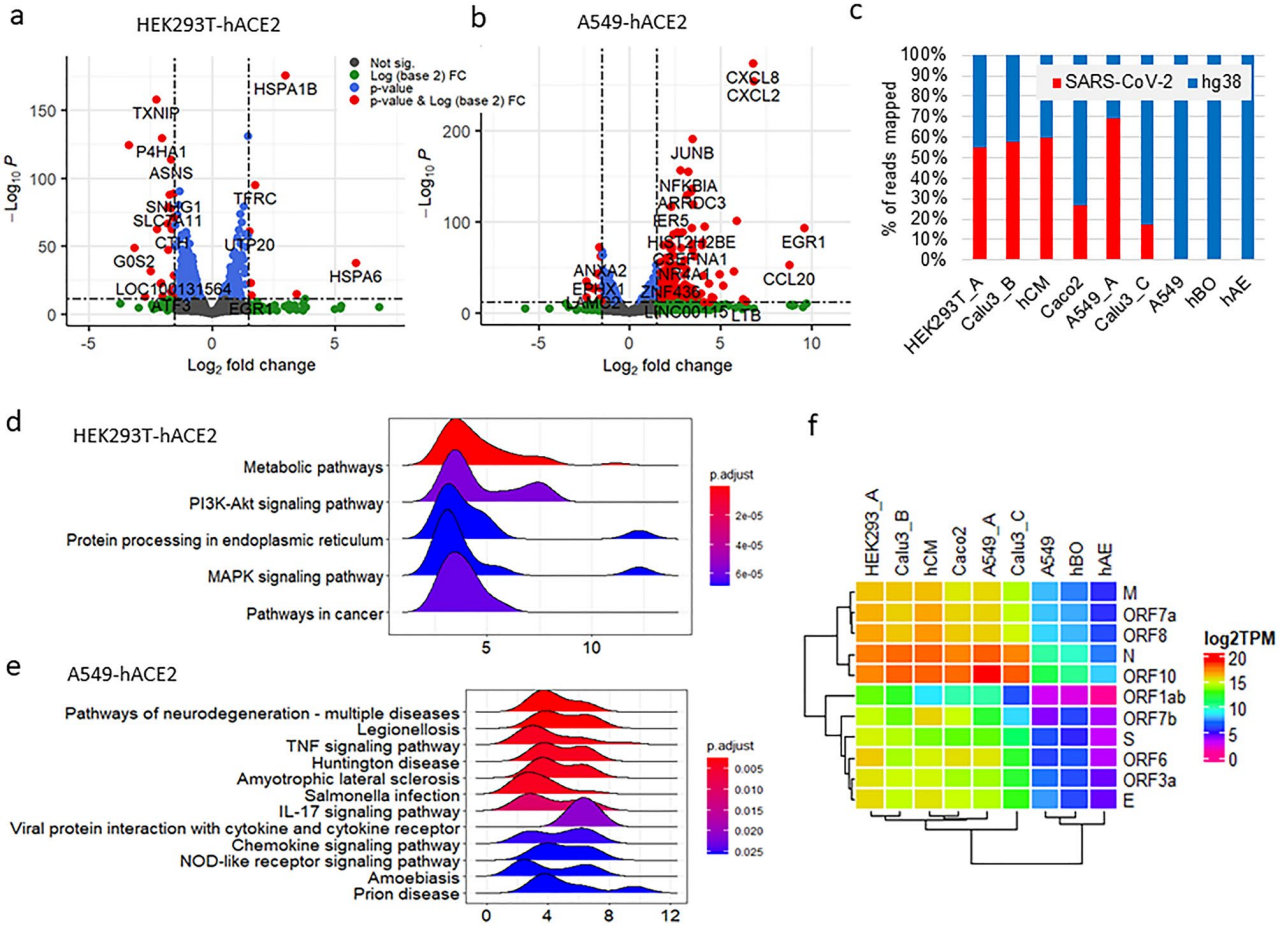
The transcriptome of SARS-CoV-2 infection in HEK293T-hACE2 cells. (**a**) Volcano Plot of DEGs of SARS-CoV-2 infection in HEK293T-hACE2 cells. (**b**) Volcano Plot of DEGs of SARS-CoV-2 infection in A549-hACE2 cells. (**c**) Percentage of reads mapped to SARS-COV-2 genome and human hg38 genome from the nine RNAseq datasets of SARS-COV-2 infection in cell lines used in this study. (**d**) Major KEGG pathways enriched with DEGs of SARS-CoV-2 infection in HEK293T-hACE2. (**e**) Major KEGG pathways enriched with DEGs of SARS-CoV-2 infection in A549-hACE2. (**f**) Heatmap of SARS-CoV-2 gene expression SARS-CoV-2 infected cells by transcripts per million (TMP). HEK293T_A and A549_A represent HEK293T or A549 with hACE2 overexpression, respectively. Calu3_B and Calu3_C represent data from the RNAseq data by Wyler E. (bioRxiv) and Blanco-Melo D. (Cell), respectively.

HEK293T cells have been used to screen SARS-CoV-2 entry factors and study the proteome, interactome, and phosphoproteome of SARS-CoV-2 infection^21,34^. However, due to very low level expression of both hACE2 and transmembrane protease serine 2 (TMPRSS2), two major host factors that mediate SARS-CoV-2 cellular entry^35,36^, HEK293T cells exhibit a very low rate of infection by SARS-CoV-2. Overexpression of hACE2 in HEK293T cells led to robust infection by SARS-CoV-2 (Fig. 1a). RNA-seq of mock-infected and SARS-CoV-2-infected HEK293T-hACE2 cells revealed several hundred significantly dysregulated host genes. As expected, we did not find any host immune response genes among the top DEGs, such as cytokines, chemokines, or interferon stimulated genes (Fig. 2a, supplemental file 2 of DEseq2 results).

Like HEK293T cells, A549 cells also have very low level of hACE2 expression and overexpression of hACE2 in A549 (A549-hACE2) cells led to robust infection^27^. In both SARS-CoV-2-infected HEK293T-hACE2 and A549-hACE2 cells, there are over 50% of mapped sequence reads aligned to SARS-CoV-2 genome, but host DEGs in these two cell lines are very different, having only 2.5% in common, with highly upregulated immune response genes only observed in A549-hACE2 cells (Figs. 1b, 2a–c). A list of DEGs in SARS-CoV-2-infected HEK293T-hACE2 cells were validated by quantitative RT-PCR (qRT-PCR) (Fig. 1c).

KEGG pathway analysis revealed that SARS-CoV-2 infection in HEK293T-hACE2 and A549-hACE2 cells triggered different pathways. The most significantly regulated pathway in SARS-CoV-2-infected HEK293T-hACE2 cells is the metabolic pathway, whereas the TNF, IL17, cytokine, chemokine, and the nucleotide-binding oligomerization domain-like (NOD-like) receptor signaling pathways are among the top upregulated pathways in the infected A549-hACE2 cells (Fig. 2d,e). Gene ontology (GO) analysis revealed that many of the DEGs in SARS-CoV-2-infected HEK293T-hACE2 cells are involved in cellular response to unfolded proteins, response to ER stress, and chaperone cofactor-dependent protein refolding, whereas many of the DEGs in SARS-CoV-2-infected A549-hACE2 cells are involved in the regulation of apoptotic signaling pathway, rhythmic process, and cellular response to biotic stimulus (Fig. S1-3). These data indicate that HEK293T-hACE2 and A549-hACE2 have a different host transcriptome upon SARS-CoV-2 infection although both have hACE2 overexpression and can achieve robust SARS-CoV-2 infection.

**Figure 3:**
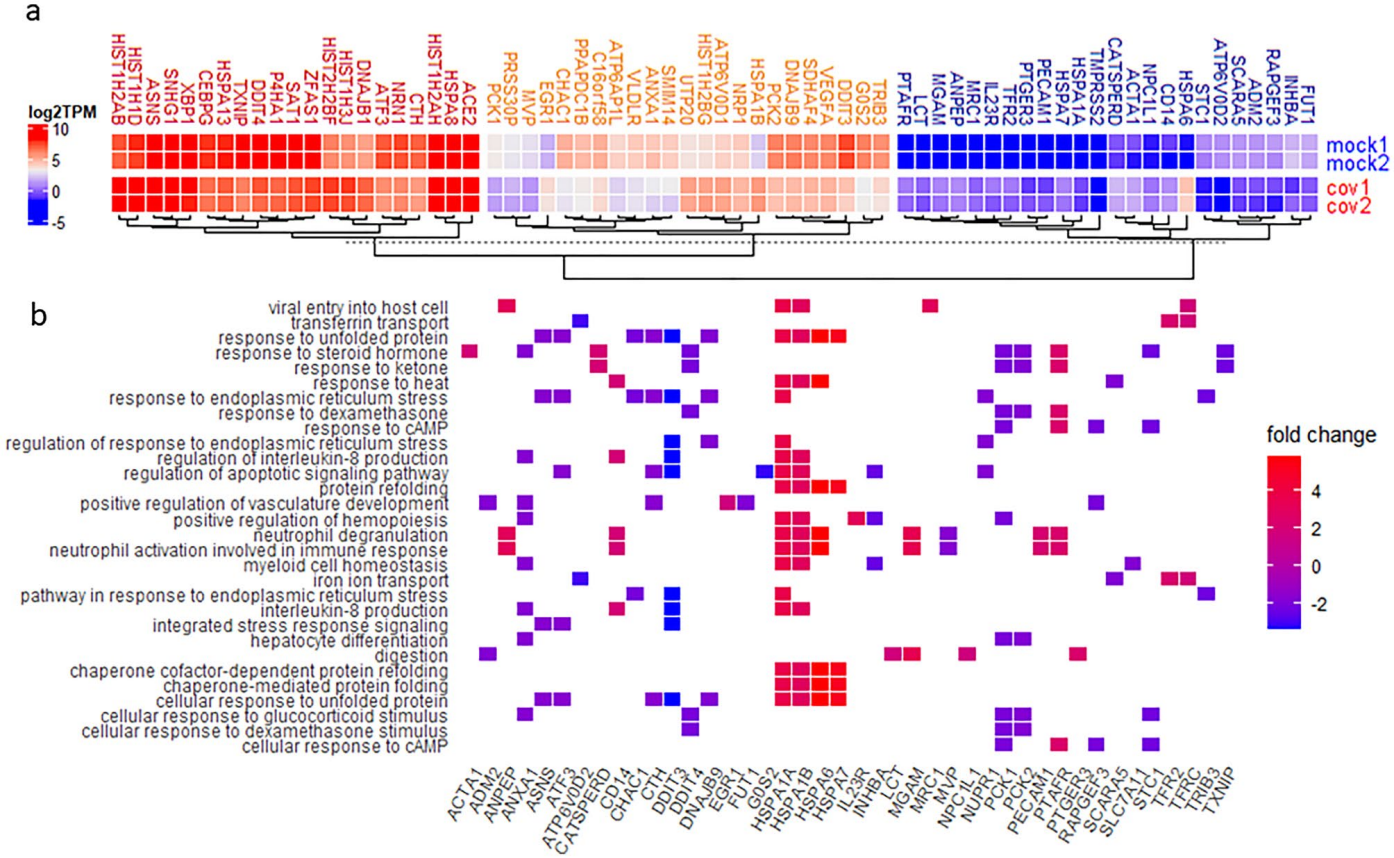
DEGs of SARS-CoV-2 infection of HEK293T-hACE2 cells. (**a**) Heatmap of DEGs in mock or SARS-CoV-2 infected HEK293T-hACE2 cells. Mock1 and mock2 indicate two replicates of mock infection and cov1 and cov2 indicate two replicates of SARS-CoV-2 infections. (**b**) Heatmap view of GO-BP enriched by DEGs from mock or SARS-CoV-2 infected HEK293T-hACE2 cells.

DEGs in HEK239T-hACE2-infected cells were clustered into three groups based on the expression level measured by transcripts per million reads (TPM). The SARS-CoV-2 host entry factors, ACE2, neuropilin-1(NRP1), and TMPRSS2, were added to the DEG list for comparison purpose, and they were clustered with genes in the high, medium, and low level of expression groups, respectively (Fig. 3a)^36–38^. Many DEGs are involved in the top 30 GO biological processes (GO-BP) (Fig. 3b).

Among the significantly upregulated DEGs, many are heat shock protein-encoding genes, such as heat shock 70 kDa protein 6 (HSPA6) and heat shock 70 kDa protein 1B (HSPA1B). These heat shock proteins are likely used by SARS-CoV-2 to mediate the folding of newly translated viral proteins. In response to SARS-CoV-2 infection, host molecular chaperones of DnaJ homolog subfamily B (DNAJB) members, DNAJB1 and DNAJB9, were also upregulated. Among genes that are significantly downregulated in SARS-CoV-2-infected HEK293T-hACE2 cells, the tribbles homolog 3 (TRIB3) gene can be induced by NF-κB and negatively regulates NF-κB expression by a feedback mechanism. TRIB3 makes cells sensitive to TNF- and TRAIL-induced apoptosis and has been reported to decrease expression during aging^39^. The thioredoxin-interacting protein (TXNIP) gene plays an important role in redox regulation and is a marker gene for diabetes that responds to glucose metabolism and inflammation^40^. The asparagine synthetase gene (ASNA) and the G0/G1 Switch Gene 2 (G0S2) are involved in cell cycle control. Activating transcription factor 3 (ATF3), usually induced during physiological stress, is a member of the cAMP-responsive element-binding protein family^41^. DNA damage-inducible transcript 3 (DDIT3) is a pro-apoptotic transcription factor that has an important role in cellular stress response by blocking the binding of C/EBP members to DNA^42^. DDIT4 is a negative regulator of mTOR that regulates cell growth, proliferation, and autophagy^43^.

These DEGs are mainly enriched in genes involved in response to UPRs and ER stress, including chaperons and cofactors, apoptotic signaling pathways as shown in gene network, GO-BP, GO cellular components (GO-CC), and GO molecular function (GO-MF) analysis. KEGG disease-related pathway analysis revealed that SARS-CoV-2 infection in HEK293T-hACE2 cells mainly affects metabolic pathways and can lead to several disease conditions, such as severe sepsis, anoxia, autoimmune disease, hyperlipidemia, Alzheimer disease, and immunodeficiency (Fig. S2).

The difference in the transcriptional response to SARS-CoV-2 infection between HEK293T-hACE2 and A549-hACE2 cells prompted us to further compare our dataset of HEK293T-hACE2 with several additional RNAseq datasets that have been deposited into GEO, including primary cells such as human pluripotent stem cell-derived cardiomyocytes (hCM)^23^, primary human airway epithelial cells (hAE)^44^, and human lung bronchial organoid cells (hBO)^45^ (Table 1, Fig. 2c). The activation of many host immune response genes upon SARS-CoV-2 infection has been reported in cells, including cell lines such as Caco2^46^, A549-hACE2^27^ and Calu3 (Calu3_B^46^ refer to dataset from the bioRxiv preprint and published on iScience by Wyler et al. ^26^, Calu3_C refer to dataset from the Cell paper by Blanco-Melo et al. ^27^) cells, and primary cells such as hCM^23^, hAE^44^, and hBO^45^ (Fig. 2f).

**Table 1:** RNAseq datasets from GEO database.

Datasets	Infection duration (hours)	MOI	% of viral reads	GSE ID
HEK293T-hACE2	72	1	54.77	169,158
A549-hACE2^27^	24	0.2	69.21	147,507
hCM^23^	72	0.1	59.58	150,392
Calu3_B^46^	12	0.3	59.59	148,729
Caco2^46^	12 and 24	0.3	26.43	148,729
Calu3_C^27^	24	2	17.39	147,507
A549^27^	24	2	0.10	147,507
hBO^45^	120	5.0 × 10^4^ PFU/well	0.06	150,819
hAE^44^	48	0.25	0.01	153,970

**Figure 4:**
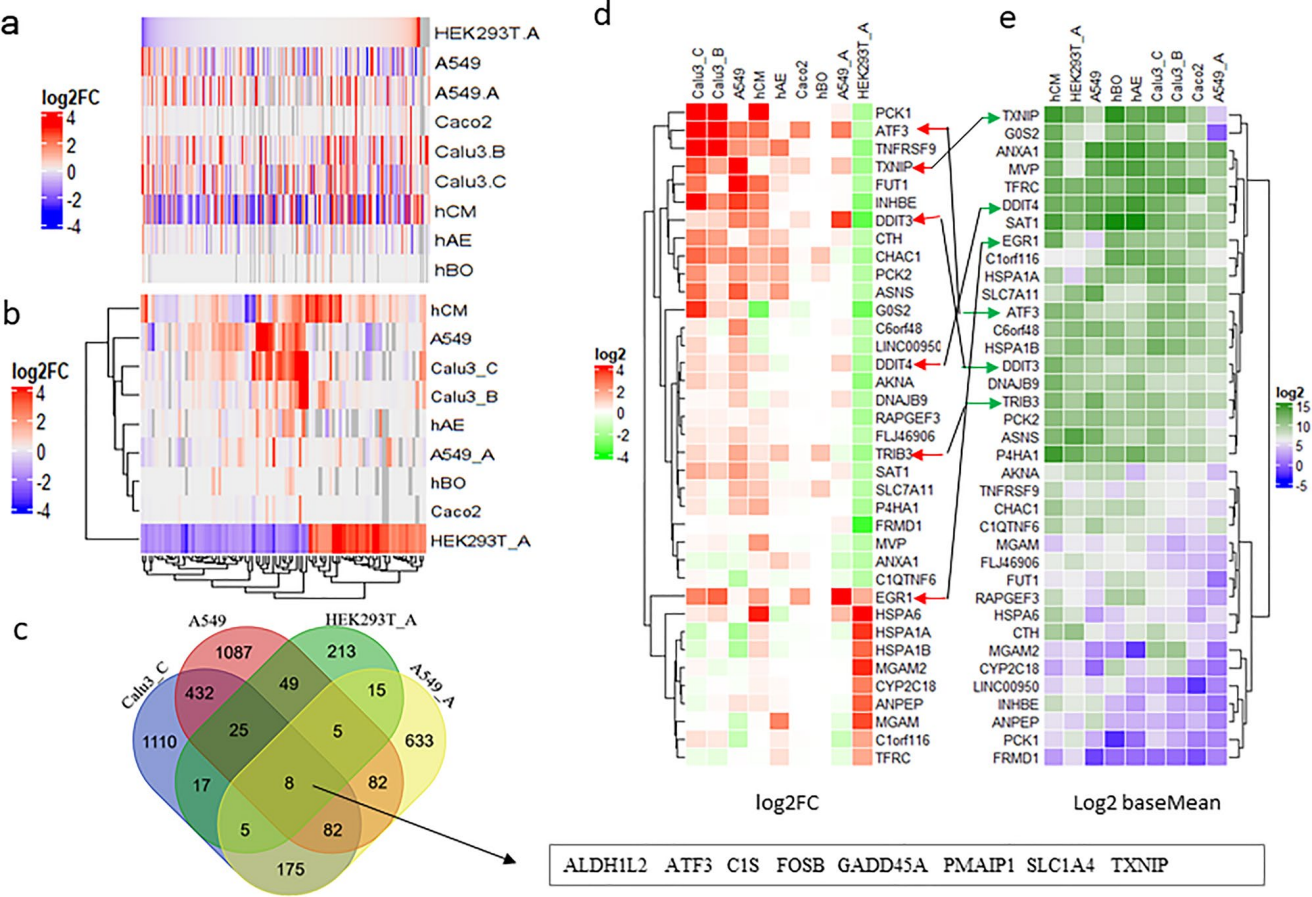
Common DEGs and genes of interest. (**a**) Heatmap view of the log2FC value of all genes in SARS-CoV-2 infected HEK293T-hACE2 cells versus those in other infected cell lines. Grey color indicates that the log2FC value are not available in the cell lines. (**b**) Heatmap view of genes with the log2FC value ≥ 2 or log2FC ≤ -1.5 in SARS-CoV-2 infected HEK293T-hACE2 cells versus those in other infected cell lines. Grey color indicates the log2FC value is not available in the cell lines. (**c**) Venn Diagram showing 8 common DEGs of infection in HEK293T-hACE2, A549, A549-hACE2, and Calu3_C cells. (**d**) Heatmap view of log2FC value of genes of interest. (**e**) Heatmap view of baseMean value (mean gene expression level calculated by DEseq2) of genes of interest. HEK293T_A/HEK293T.A and A549_A/A549.A represent HEK293T or A549 with hACE2 overexpression, respectively. Calu3_B/Calu3.B and Calu3_C/Calu3.C represent data by Wyler E. (bioRxiv) and Blanco-Melo D. (Cell), respectively.

To compare gene expression profile of SARS-CoV-2-infected HEK293T-hACE2 cells with other infected cells, the log2 fold changes (log2FC) value of all genes from these infected cells were sorted in descending order according to the value in HEK293T-hACE2 cells before generating a heatmap. As shown in the heatmap, many of the genes that are altered upon SARS-CoV-2 infection in other cell types did not exhibit substantial expression change in infected HEK293T-hACE2 cells (Fig. 4a).

Next, we selected a list of DEGs in HEK293T-hACE2 cells with log2FC ≥ 2 and log2FC ≤ -1.5 (we used 1.5-fold as the cut-off for down-regulated genes to increase the number of downregulated genes in order to balance the DEG list because there are more genes in the upregulated list) and false discovery rate (FDR, *P*-adj value in DEseq2 results) < 0.05, and then compared to their log2FC values in other data sets (Fig. 4b). As shown in the heatmap, many of the significantly dysregulated genes in SARS-CoV-2-infected HEK293T-hACE2 cells were absent in other infected cell lines.

By comparing DEGs with absolute value of log2FC [abs(log2FC)] ≥ 1 and FDR < 0.05 in A549, A549_hACE2, Calu3_C, and HEK293T-hACE2 cells datasets, we identified an eight-gene signature that is common to the datasets from SARS-COV-2-infected cells of different tissue types and with different host immune response status (Fig. 4c). These eight genes may represent non-immune genes that are commonly altered upon SARS-CoV-2 infection. The unique host transcriptome of SARS-CoV-2 infection in HEK293T-hACE2 cells allowed us to identify genes and pathways regulated by viral infection beyond host immune response.

### Identifying host response factors beyond immune response genes

It is conceivable that there are a number of host transcriptomic, epigenomic, and proteomic changes associated with the acute and long-term symptoms of COVID-19 that have been masked by the extensive host immune response in severe cases. Therefore, we set to identify major factors beyond those mediating the host immune response by comparing data from SARS-CoV-2-infected HEK293T-hACE2 cells that are defective of host immune response with the datasets from cells with robust host immune response.

We focused on a list of 37 genes based on DEGs that have abs(log2FC) ≥ 2, baseMean (mean gene expression reported in DEseq2 results) value over 20, and FDR below 10^–5^ (Fig. 4d,e). We found that three heat shock genes, *HSPA6, HSPA1A*, and *HSAP1B,* were highly upregulated in infected HEK293T-hACE2 cells, but downregulated in the rest of infected cells analyzed except in hCM cells. It is interesting that the downregulated genes in SARS-CoV-2-infected HEK293T-hACE2 cells were either upregulated or unchanged in other infected cells. There is only one exception that the early growth response proteins (EGR) encoding gene 1, *EGR1*, which was recently identified as an essential factor for SARS-CoV-2 infection by a pooled genome-wide CRISPR-Cas9 knockout screen^47^, stands out in that it is upregulated in all infected cells with high viral load. Among genes that were upregulated in infected cells with high viral load (Calu3_B, Calu3_C, A549-hACE2, and hCM), we found three members of EGRs, *EGR1, EGR2*, and *EGR3*, that were present in the common gene list with genes of cytokines, genes involved in NF-κB, MAPK, TNF pathways, further supporting a critical role of EGRs in SARS-CoV-2 infection (Fig. S4).

Next, we looked at the 10 DEGs that were commonly induced in infected Calu3_C, A549, hAE, and hBO, regardless of the viral load. We looked at their presence in other RNAseq data that have been curated in coronavirus RNAseq database by Metascape^48^. Metascape analysis showed that they are present in the upregulated gene list of several RNAseq datasets and these genes are involved in inflammation and inflammatory disorder (Fig. S5a-b). Next, we combined these 10 genes with the 8 common genes from HEK293T-hACE2, A549, A549-hACE2 and Calu3_C. Most of these genes (14 of 18) are present in RNAseq samples as upregulated genes (Fig. S5c). There is a total of 362 curated datasets (including 125 patient samples) of host transcriptome, phosphoproteome, interactome, ubiquitinome, proteome, and translatome in coronavirus database by Metascape. The *TXNIP* gene that was downregulated in SARS-CoV-2-infected HEK293T-hACE2 cells was present in 30 of the 125 patient samples, all of which are RNASeq datasets (Figs. 1c, 2a, S5d). Therefore, TXINP may play a role in COVID-19 development and its therapeutic potential deserve further study.

### Human ACE2 expression and host immune reaction to SARS-CoV-2 infection

Interestingly, in the above reanalyzed data, we noticed that infection with very low viral load can activate some host immune response pathways similar to infection with very high viral load (Figure S3: A549-hACE2, S3: A549, S7: Calu3_C, S8: hCM, and S9: hAE). We performed similar log2FC value plot of significantly regulated genes in several samples as we did in HEK293T-hACE2 cells. Due to the high number of genes in the list, we used genes with abs(log2FC) ≥ 2 and FDR < 0.05 for A549, A549-hACE2, Calu3_B, Calu3_C, Caco2, and hCM for plotting.

First, we noticed that there are almost equal number of upregulated or downregulated genes in SARS-CoV-2-infected A549 and hCM cells, whereas most of the significantly dysregulated gene in SARS-CoV-2-infected Calu3 and Caco2 cells were upregulated. Of particular interest, data from infected A549 cells showed very low viral load (total viral reads by percentage is less than 0.01% in total reads mapped to hg38 genome or SARS-CoV-2 genome), but still triggered expression level change in a large number of genes.

Some of the highly upregulated genes in infected A549 cells were also present in the dataset of SARS-CoV-2-infected A549-hACE2 cells, Calu3, and hCM cells (Fig. S10a-f). This prompted us to compare DEGs of Calu3_C, A549, hAE, and hBO, which had low level of viral infection (Fig. 2c,f). Because SARS-CoV-2 infection caused very low level gene expression change in hBO, to have enough number of DEGs for comparison, we adjusted the abs(log2FC) cutoff for hBO to 0.7 (genes with over 1.5-fold change) and FDR < 0.05 to get its DEG list (Fig. S10g).

**Figure 5:**
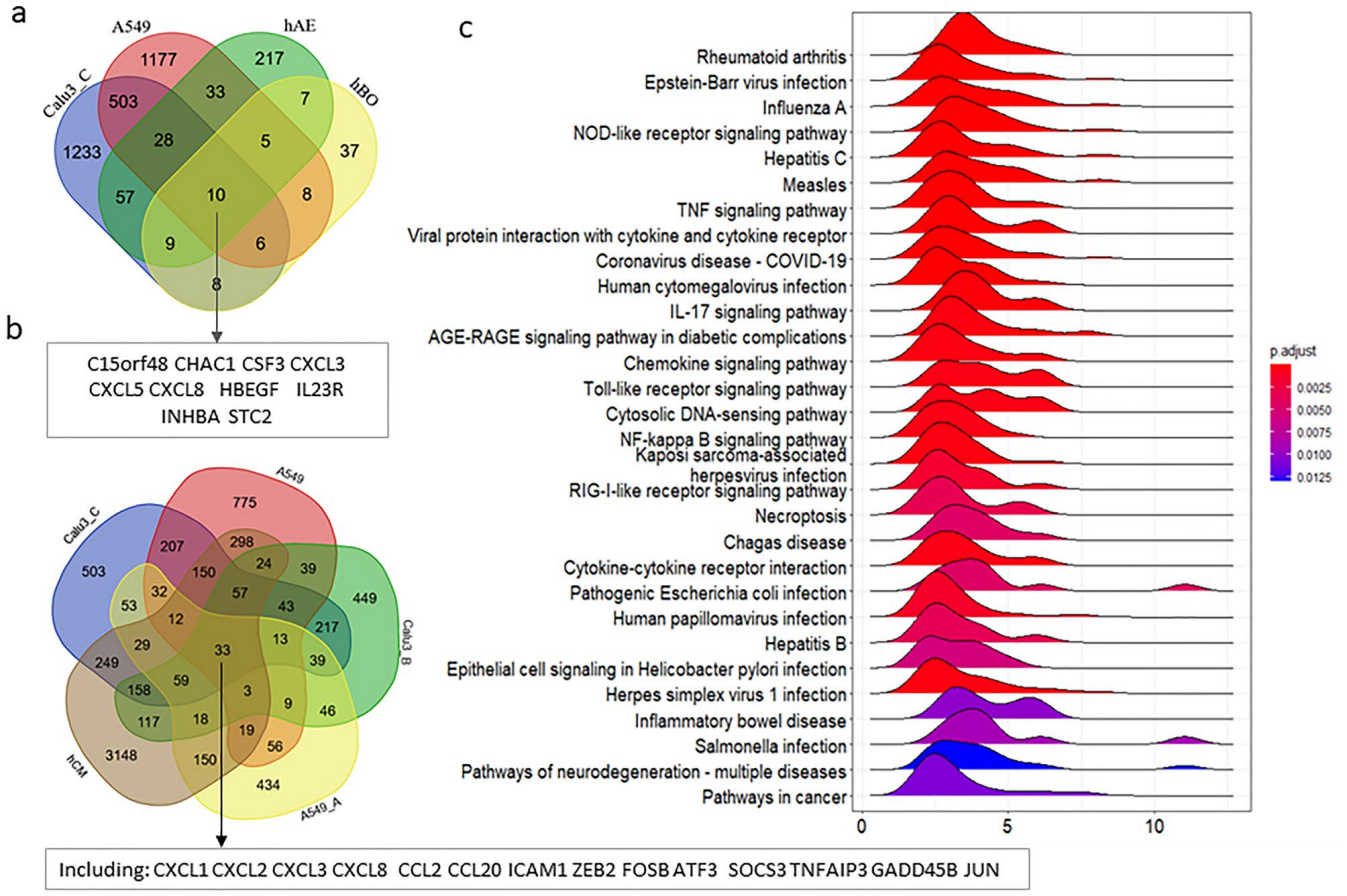
hACE2 expression and SARS-CoV-2 infection. (**a**) A Venn Diagram showing the 10 common DEGs in the 4 samples with low level of infection, including A549, hAE, hBO, and Calu3_C cells. (**b**) A Venn Diagram showing the partial list of common DEGs in the 2 samples with low level of infection (A549 and Calu3_C cells) and 3 samples with high level of infection (A549-hACE2, hCM, and Calu3_B cells). (**c**) Top 30 KEGG pathways enriched with DEGs of SARS-CoV-2 infection in Calu3_C cells. A549_A represents A549 with hACE2 overexpression. Calu3_B and Calu3_C represent data by Wyler E. (bioRxiv) and Blanco-Melo D. (Cell), respectively.

We found that the immune response genes *CXCL3, CXCL5,* and *CXCL8* are among the 10 common genes in these four cell types (Fig. 5a). Comparing DEGs of infected A549 cells (low viral load) with DEGs of infected Calu3, hCM, and A549-hACE2 cells (high viral load), we found the immune response genes *CXCL1, CXCL2, CXCL3, CXCL8, CCL2,* and *CCL20* are among the 33 DEGs common to these cell lines (Fig. 5b).

These data indicate that even very low viral load can trigger host immune response by activating the host cytokine and chemokine-related pathways. Although there are more immune response genes that are activated in SARS-CoV-2-infected A549-hACE2 cells than that in infected A549 cells, there is not a direct correlation between the extent of host immune response and the level of viral load when comparing infected A549-hACE2 cells with infected Calu3_C cells (Fig. S10). There was a much broader spectrum of immune response in infected Calu3_C cells than that in infected A549-hACE2 cells even though infected Calu3_C cells only had about 1/5 viral load of that in infected A549-hACE2 cells (Fig. 2c). Like infected hCM, Calu3_B cells, and Caco2 cells, infected Calu3_C cells activated many of the cellular response, of which defense response to type I interferon, type I interferon signaling pathways, response to viral infection, and activation of interferon response-related pathways are among the top 5 GO categories (Fig. 5c, S7).

**Figure 6:**
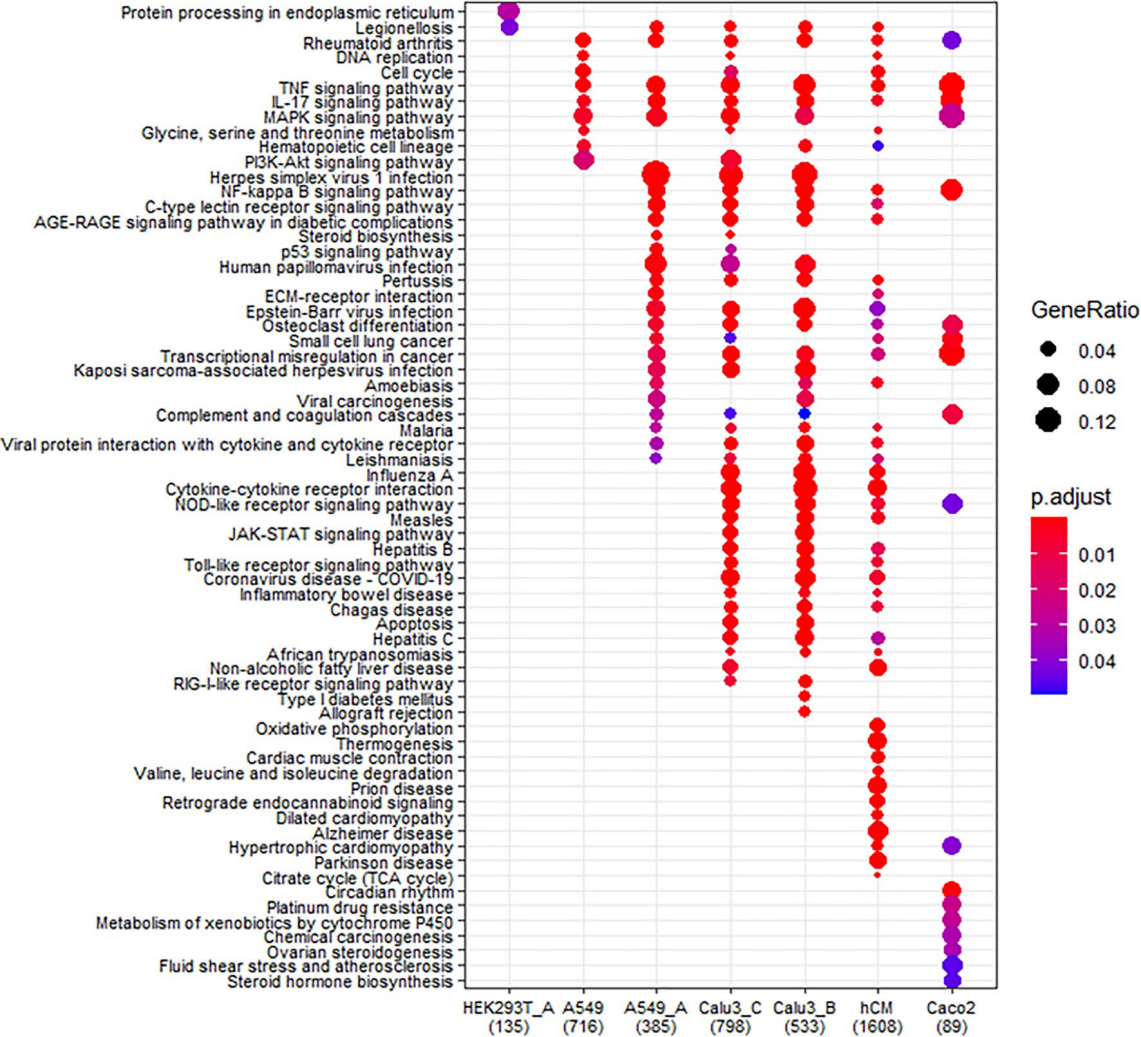
Comparison of GO enrichment analyses of DEGs with abs(log2FC) ≥ 1 and FDR < 0.05. DEGs in HEK293T-hACE2 (HEK293T_A), A549, A549-hACE2 (A549_A), Calu3_B, Calu3_C, hCM, and Caco2 cells. Calu3_B and Calu3_C represent data by Wyler E. (bioRxiv) and Blanco-Melo D. (Cell), respectively.

To compare the difference at pathway level, we compared GO categories in cells with low viral load (A549) with that in cells with high viral load and hACE2 overexpression (A549-hACE2 and HEK293T-hACE2), cells with high viral load and high level of endogenous ACE2 expression (Calu3_B and hCM), and cells with medium viral load and high level of endogenous ACE2 expression (Calu3_C and Caco2) (Figs. 2c, 6; Table 1).

Regardless of viral load, SARS-CoV-2 infection triggered the activation of TNF, IL-17, and MAPK signaling pathways in all cells except HEK293T-hACE2. A549 and Calu3_C also triggered the PI3K-AKT signaling pathway that controls cell survival and proliferation. This pathway was shut down when viral load reached higher level in Caco2, A549-hACE2, Calu3_B, and hCM cells. These data indicate that TNF, IL-17, and MAPK signaling pathways that are proinflammatory and can lead to cell apoptosis and symptoms such as asthma and fever, are very sensitive to SARS-CoV-2 infection and can produce the earliest immune response factors when the infection is still at early stage and the viral load is still low.

When the viral load reached higher level, SARS-CoV-2 infection triggered several virus-induced immune reactions and NF-kB pathway in the host. Of interest, it also triggered the AGE-RAGE (advanced glycation end products-receptor of advanced glycation end products) signaling pathway, a pathway that has been shown to be involved in diabetic complications, pulmonary inflammatory responses, and cognitive impairment^49–51^. However, the Influenza A, Hepatisis B and C, and Measles-like immune reaction was only activated in naturally permissive cells with high viral load when compared to A549-hACE2 infection. In cells that express high level of endogenous hACE2, cytokine-cytokine receptor interaction, NOD-like receptor JAK-STAT, Toll-like receptor, and RIG-I-like signaling pathways were activated upon SARS-CoV-2 infection. The interferon-induced genes, such as *OAS* genes, *IFIT* genes, *INFB* genes, were only up-regulated in the three highest viral load datasets with high endogenous hACE2 expression, Calu3_B, Calu3_C, and hCM, but not in A549-hACE2 cells in which hACE2 was overexpressed ectopically. These data indicate that once viral load peaked in cells that express high level of endogenous hACE2, it will trigger the activation of cytokine response, response to exogeneous dsRNA, activation or reinforcing JNK, NF-κB, MAPK, JAK-STAT signaling pathways, then the infected cells will mount a full spectrum of immune response, such as cytokine release syndrome that can lead to severe COVID-19 symptoms (Figs. 6, S11 ).

## Discussion

Despite it has now been over a year since the start of the COVID-19 pandemic, many key questions about SARS-CoV-2 infection and different COVID-19 symptoms it causes remain unanswered. An intriguing question about COVID-19 is how SARS-CoV-2 interplays with host during infection and how SARS-CoV-2 infection can cause so many disease symptoms. The COVID-19 symptom study app has documented over 60 symptoms and around 20% recovered patients have experienced long COVID symptoms. However, there are around 80% SARS-CoV-2 infection that does not lead to symptoms or only cause mild flu-like symptoms. Mechanisms underlying the development of chronic post-COVID-19 syndrome also remain largely unknown.

The transcriptome of SARS-CoV-2-infected cells that reflects the interplay between host and virus has provided valuable insights into mechanisms underlying SARS-CoV-2 infection and COVID-19 disease progression. However, these published works showing change in host transcriptome or proteome have been mainly focused on samples with acute and severe symptoms caused by SARS-CoV-2 infection and pointed to the dysregulated host immune reactions as the major cause for the severe outcome of SARS-CoV-2 infection. In the current research, we reported an “asymptomatic” like transcriptome of SASR-CoV-2 infection in HEK293T-hACE2 cells, in which SARS-CoV-2 infection activated many ER stress-related UPR pathways instead of mounting a substantial immune response to the SASR-CoV-2 infection due to the defect of innate immunity in HEK239T cells. The dysregulated UPR could be one of the major causes of COVID-19 symptoms besides host immune response because ER stress could lead to aberrant metabolism, organelle dysfunction, and inflammation^52^. Such pathways are masked by the overwhelming host immune response in cell types that exhibit extensive host immune response. Single cell RNAseq in limited number of COVID-19 patients with asymptomatic, moderate, or severe conditions identified activation of immune response genes as a prominent feature of severe COVID-19, a response that distinguishes the disease outcome from severe to mild or asymptomatic in infected individuals^31^. Therefore, the induction of genes and pathways other than the immune response we detected in HEK293T-hACE2 cell matches the gene expression profile in COVID patients who are asymptomatic or with mild symptoms , both of which lack severe immune response.

Our data showed that very low level of viral infection can also trigger a large-scale host immune response and lead to dysregulation of hundreds and thousands of genes, suggesting that the COVID-19 disease status may not correlate well with the viral load in an infected individual. This hypothesis can be supported by the result showing that the viral load within asymptomatic individuals was indistinguishable from that in symptomatic individuals^53^.

Our analysis of different datasets supports the notion that hACE2 expression level is directly correlated with the viral load but not with the scale of the immune response. We found that there is a clear difference in response to SARS-CoV-2 infection in naturally permissive cells with high level of endogenous ACE2 expression and not very permissive cells with low level of endogenous ACE2 expression. Only naturally permissive cells with high endogenous ACE2 expression mount a full-scale immune attack in response to SARS-CoV-2 infection. In this sense, it is conceivable that the type of tissues that are initially infected by SARS-CoV-2 in COVID-19 patients may impact the magnitude of the immune response, thus the extent of disease progression. Therefore, the infection route may be critical for the severity of COVID-19 disease . It is expected that infection by SARS-CoV-2 through routes with high number of virally permissive cells, such as the alveolar epithelial cells in the upper airway of respiratory system that have high level of ACE2 expression, could lead to COVID-19 progression to severe symptoms faster. On the other hand, infection through cells in the eyes, gastrointestinal tract, or reproductive system may need longer incubation time for the progression of disease and the onset of symptoms, which could explain why the development of COVID-19 have a wide range of incubation time and symptoms^54^. The difference in the extent of the immune response between highly permissive and slightly permissive cells suggests that once the full-scale immune response is triggered by highly permissive cells, irreversible and severe damage to cells and tissues can occur, consequently patients can progress into the severe state. Hence, antiviral drugs may not help severely ill patients, but early antiviral intervention in patients before reaching the severe state may have protective effects by blocking or slowing down viral spreading into tissues with highly permissive cells.

HEK293T cells have been widely used in SARS-CoV-2 studies, including screening for SARS-CoV-2 entry factors, replication factors, generating the infection proteome, interactome, phosphoproteome, and testing antiviral drugs. The current study indicates that those studies address situations without host immune response, thus interpretation of those results need to take the immune response into consideration.

## Materials and method

### Cell lines

Vero-E6 cell line (ATCC CRL1586) and HEK293T (ATCC CRL-11268) cell lines were obtained from the American Type Culture Collection (ATCC).

The hACE2-HEK293T cells were made by transient transfecting HEK293T cells with hACE2 expressing vector (Addgene #1786) using calcium phosphate precipitation method, and the cells were subjected to SARS-CoV-2 infection, and RNA preparation for RNA-seq.

All cells were cultured in DMEM growth media (10% fetal bovine serum, 2 mM L-glutamine, penicillin (100 units/ml), streptomycin (100 units/ml), and 10 mM HEPES), and cultured at 37 °C and 5% CO_2_.

### SARS-CoV-2 infection, expansion, and quantification of viral RNA

SARS-CoV-2 related works, such as virus expansion and quantification of viral RNA were performed as previously described^23,55^. SARS-CoV-2 isolate USA-WA1/2020, was obtained from the Biodefense and Emerging Infections (BEI) Resources of the National Institute of Allergy and Infectious Diseases. All procedures involving SARS-CoV-2 infection were conducted within a Biosafety Level 3 facility at UCLA. SARS-CoV-2 was passaged once in Vero-E6 cells and viral stocks were aliquoted and stored at -80 °C. Virus titer was measured in Vero-E6 cells by TCID50 assay. For SARS-CoV-2 infection of HEK293T-hACE2 cells, viral inoculum (MOI of 1, or 1 plaque forming unit per cell) was prepared using serum-free medium. Culture medium was removed and replaced with 250 μL of prepared inoculum in each well. For mock infection, serum-free medium (250 μL/well) alone was added. The inoculated plates were incubated at 37 °C with 5% CO_2_ for 1 h. The inoculum was spread by gently tilting the plate sideways at every 15 min. At the end of incubation, the inoculum was replaced with fresh medium. Cells remained at 37 °C with 5% CO_2_ for 72 h. At the end of incubation, Trizol reagent (Thermo Fisher) was added to the well for total RNA isolation.

### RT-qPCR and statistical analysis for qPCR data

RT-qPCR analysis was performed as described^55^. Specifically, Complementary DNA (cDNA) was synthesized using Tetro cDNA Synthesis Kit (Thomas Scientific). qRT-PCR was performed using SYBR Green Master Mix (Thermo Scientific) on the Step One Plus Real-Time PCR Instrument (Applied Biosystems). *ACTIN * was used as the reference gene. qPCR primers were designed using online primer design tool (https://www.ncbi.nlm.nih.gov/tools/primer-blast/). Statistical significance was analyzed using GraphPad Prism 8. Unpaired Student’s t test was used. For all test, p values were presented as ∗ *P* < 0.05, ∗∗ *P* < 0.01, and ∗∗ ∗ *P* < 0.001. Error bar stands for SD.

### RNA Sequencing (RNAseq)

Total RNA and the KAPA RNA HyperPrep Kit with RiboErase (Roche) was used for RNAseq library preparation. Sequencing was done on an Illumina HiSeq 2500 machine with a single read at 51 bp read length and coverage of ~ 30 M reads/sample.

### RNAseq data processing, analysis, and statistics

To have the data comparable, we reanalyzed the RNAseq datasets using the same procedure, the same data processing software, and the same genome for mapping and reads counting (Supplemental file1. Subread genes count.csv). We then fed the raw mapped reads count into DEseq2 to generate DEG results that include the log2 fold changes (log2FC) for all genes of infected samples versus corresponding mock infected samples (Supplemental file 2. DEseq2 resutls.csv).

Briefly, raw reads obtained from RNAseq were mapped to the human hg38 genome (iGenomes from Illumina) and SARS-CoV- 2 reference genome (GenBank: NC_045512) using subread aligner and read counting to genes were obtained with featureCounts. Subread aligner and featureCounts in Subread package release 2.0.1 were used with default parameters^56^. Integrative Genomics Viewer (IGV) 2.8 was used to view reads alignments in genome. Unnormalized read count from featureCounts were input into Bioconductor package DEseq2 (version 1.30.0) in R version 4.0.2 to generate the differentially expressed gene results (infected versus mock) at default setting [the Wald-test was applied to assess the p value for differential gene expressions and the adjusted p value (p-adj) was done by Benjamini and Hochberg method]^57^. Unless specified in the figure legends, *P*-adj < 0.05 was used as cutoff for gene expression with significant changes.

Many Linux freeware, R or Bioconductor packages were used for data analysis and visualization. Such as ComplexHeatmap for heatmaps^58^, EnhancedVolcano for volcano plots, ClusterProfiler and Enrichment plot for GO and KEGG analysis^59^. Patient related COVID-19 gene list in Coronascape was downloaded from Metascape website^48^.

## Supplementary Information

Supplementary Information 1.

Supplementary Information 2.

Supplementary Information 3.

## Data Availability

RNAseq data was deposited into GEO database under access number GSE169158. Raw gene counts from subread2 for HEK293T-hACE2 are listed in Supplemental file 1: HEK293T-hACE2_genes_count.xlsx. DEseq2 produced differential gene expression (viral infection versus mock infection) results for all datasets are listed in Supplemental file 2: DEseq2 results_condition_cov_vs_mock.xlsx.
