# Supplementary material for: Comparative transcriptomic analysis of SARS-CoV-2 infected cell model systems reveals differential innate immune responses
Source: Sci Rep. 2021 Aug 25;11:17146. doi: 10.1038/s41598-021-96462-w (PMC8387424; doi:10.1038/s41598-021-96462-w)
Supplement: Supplementary file 2 — Supplementary Information 2. [file 41598_2021_96462_MOESM2_ESM.pdf]

Supplementary information to

Comparative transcriptomic analysis of SARS-CoV-2 infected cell model systems reveals differential innate immune responses

Guihua Sun[1], Qi Cui[2], Gustavo Garcia Jr. [3,4], Cheng Wang[2], Mingzi Zhang[2], Vaithilingaraja Arumugaswami[3,4*], Arthur D. Riggs[1*], Yanhong Shi[2*]

1. qPCR primers

Primer name	Sequence (5' to 3')
TXNIP-F	GCCAGCCAACTCAAGAGACA
TXNIP-R	CCCGCCCATCAGGAATGAAC
DITT3-F	TTGCCTTTCTCCTTCGGGAC
DITT3-R	CAGTCAGCCAAGCCAGAGAA
DDIT4-F	CGAACTCCCACCCCAGATCG
DDIT4-R	GGTTGGCACACAAGTGTTCAT
G0S2-F	CCAAGGAGATGATGGCCCAG
G0S2-R	CTTGCTTCTGGAGAGCCTGT
TRIB3-F	TGCGTGATCTCAAGCTGTGT
TRIB3-R	GCTTGTCCCACAGGGAATCA
XBP1-F	ACCAGGAGTTAAGACAGCGC
XBP1-R	ACTGGCCTCACTTCATTCCC
HSPA6-F	GATGTGTCGGTTCTCTCCATTG
HSPA6-R	CTTCCATGAAGTGGTTCACGA
HSPA8-F	ACCTACTCTTGTGTGGGTGTT
HSPA8-R	GACATAGCTTGGAGTGGTTCG
SAT1-F	ACCCGTGGATTGGCAAGTTAT
SAT1-R	TGCAACCTGGCTTAGATTCTTC
HSPA1b-F	TTTGAGGGCATCGACTTCTACA
HSPA1b-R	CCAGGACCAGGTCGTGAATC

2. Supplemental figures and legends:

Figure S1. Upset plot of GO enrichment of HEK293T-hACE2 versus A549-hACE2. **a.** GO enrichment of SARS-CoV-2 infection in HEK293T-hACE2 cells. **b.** GO enrichment of SARS-CoV-2 infection in A549-hACE2 cells.

a HEK293T-hACE2

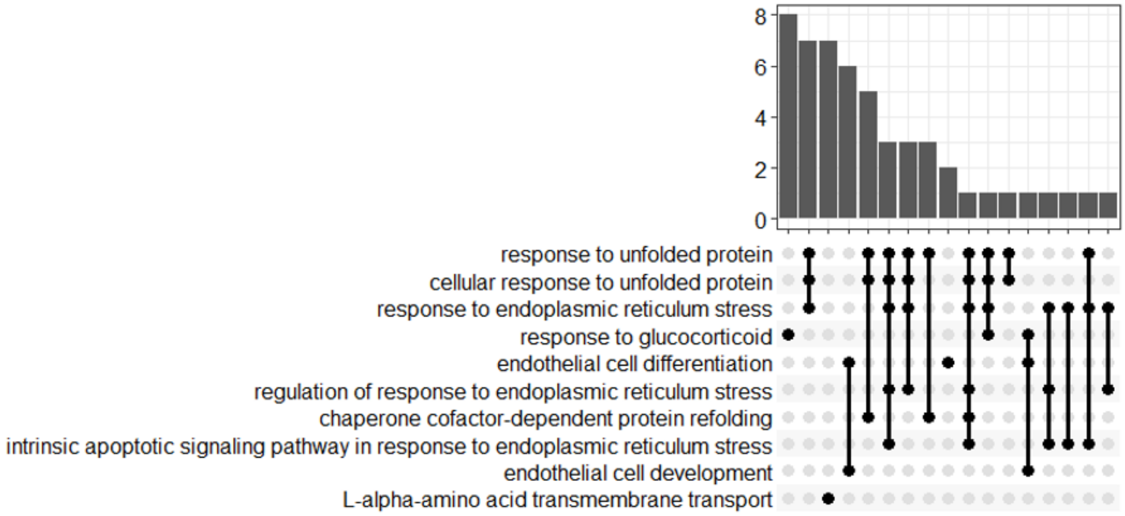

b A549-hACE2

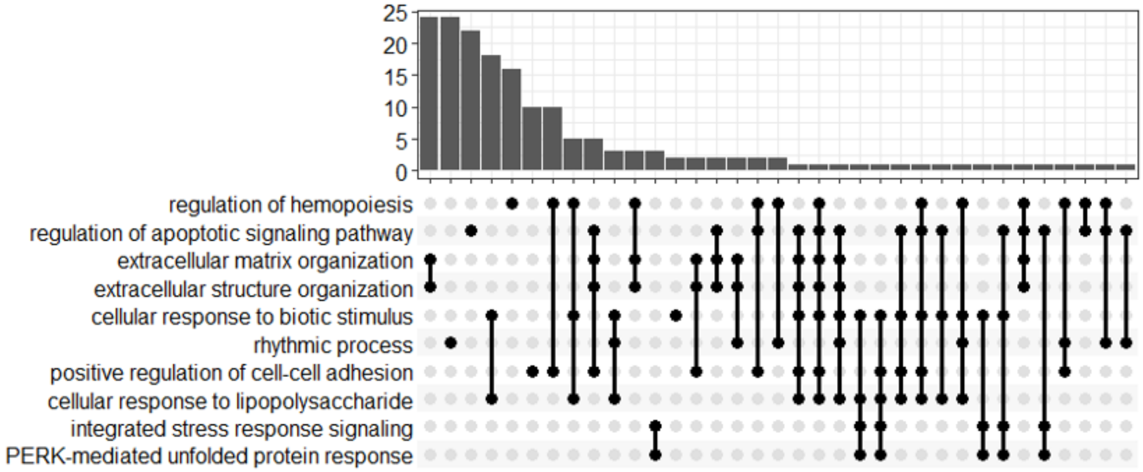

Figure S2. GO and network analysis of DEGs of SARS-CoV-2 infection of HEK293T-hACE2 cells. **a.** Network view of top 5 enriched GO-BP. **b.** Bar plot of top 20 GO-BP. **c.** Dot plot view of top 10 GO-CC and top 10 GO-MF. **d**. Bar plot view of the involvement of DEGs in the top 20 diseases.

a

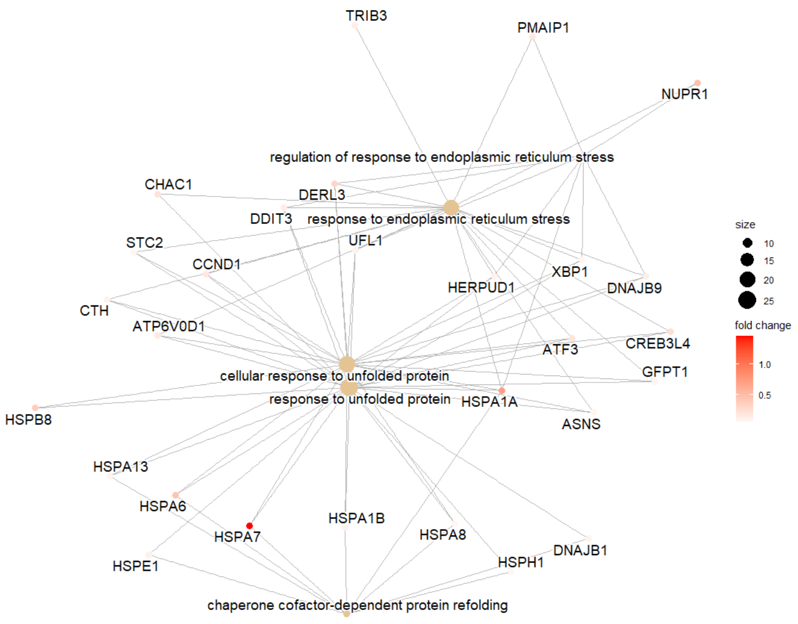

b

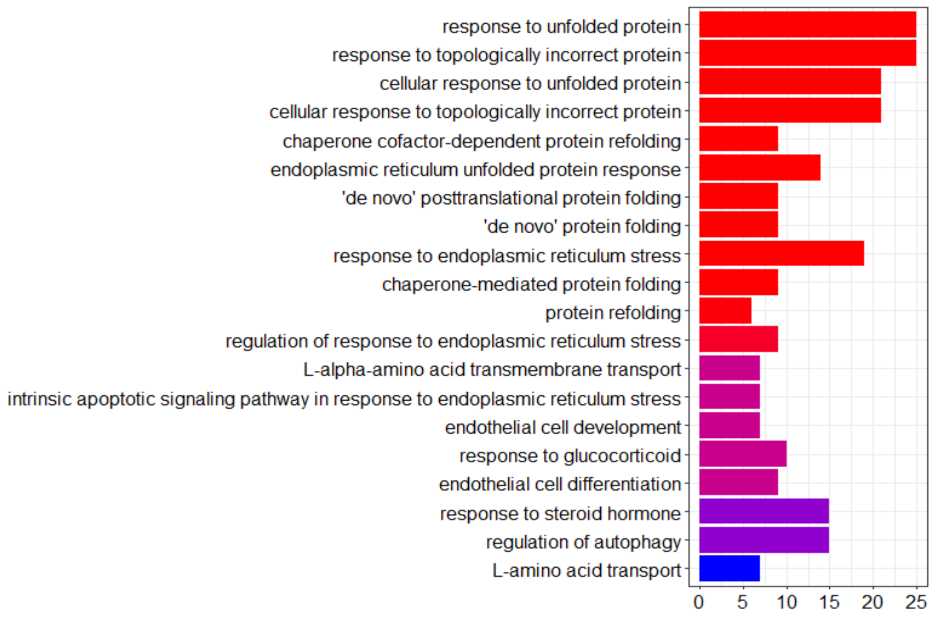

c.

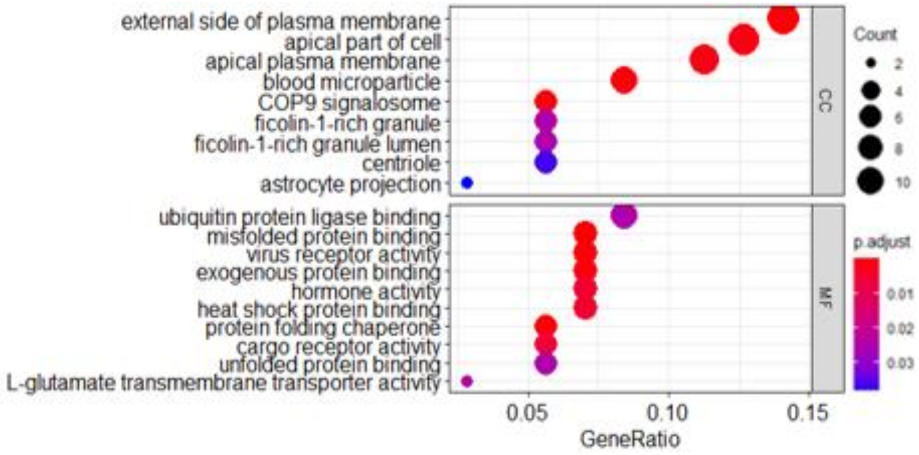
external side of plasma membrane
apical part of cell
apical plasma membrane
blood microparticle
COP9 signalosome
ficolin-1-rich granule
ficolin-1-rich granule lumen
centriole
astrocyte projection
ubiquitin protein ligase binding
misfolded protein binding
virus receptor activity
exogenous protein binding
hormone activity
heat shock protein binding
protein folding chaperone
cargo receptor activity
unfolded protein binding
L-glutamate transmembrane transporter activity
CC
MF
Count
2
4
6
8
10
p.adjust
0.01
0.02
0.03
0.05
0.10
0.15
GeneRatio

d

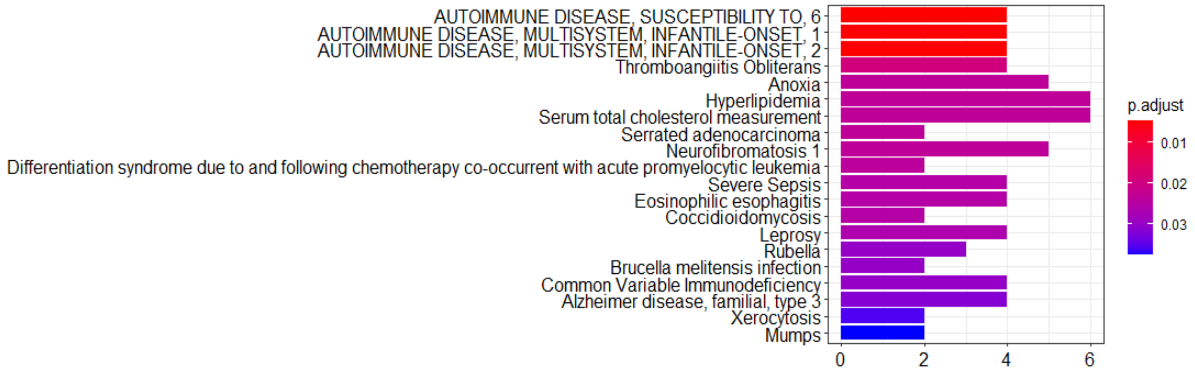
AUTOIMMUNE DISEASE, SUSCEPTIBILITY TO, 6
AUTOIMMUNE DISEASE, MULTISYSTEM, INFANTILE-ONSET, 1
AUTOIMMUNE DISEASE, MULTISYSTEM, INFANTILE-ONSET, 2
Thromboangiitis Obliterans
Anoxia
Hyperlipidemia
Serum total cholesterol measurement
Serrated adenocarcinoma
Neurofibromatosis 1
Differentiation syndrome due to and following chemotherapy co-occurrent with acute promyelocytic leukemia
Severe Sepsis
Eosinophilic esophagitis
Coccidioidomycosis
Leprosy
Rubella
Brucella melitensis infection
Common Variable Immunodeficiency
Alzheimer disease, familial, type 3
Xerocytosis
Mumps
0
2
4
6
p.adjust
0.01
0.02
0.03

Figure S3. GO and network analysis of DEGs of SARS-CoV-2 infection of A549-hACE2 cells. **a.** Dot plot of top-10 GO-BP, GO-CC, and GO-MF. **b.** Bar plot of top 20 GO-BP. **c.** Network view of the involvement of DEGs in the top 5 GO-BP categories. **d.** Bar plot view of the involvement of DEGs in the top 20 categories of disease.

a

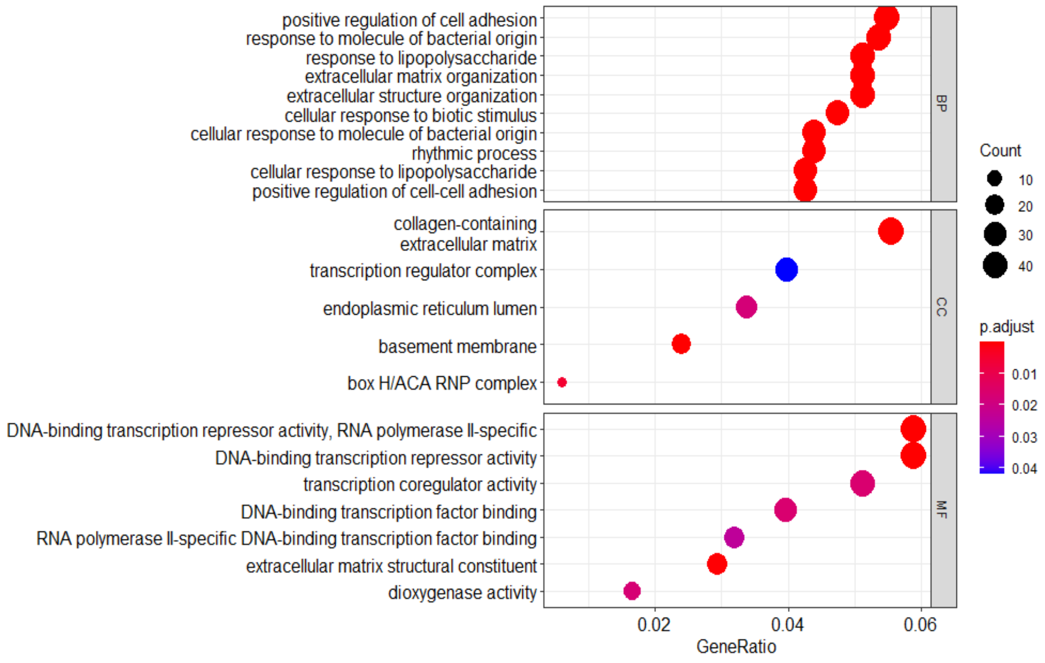

b

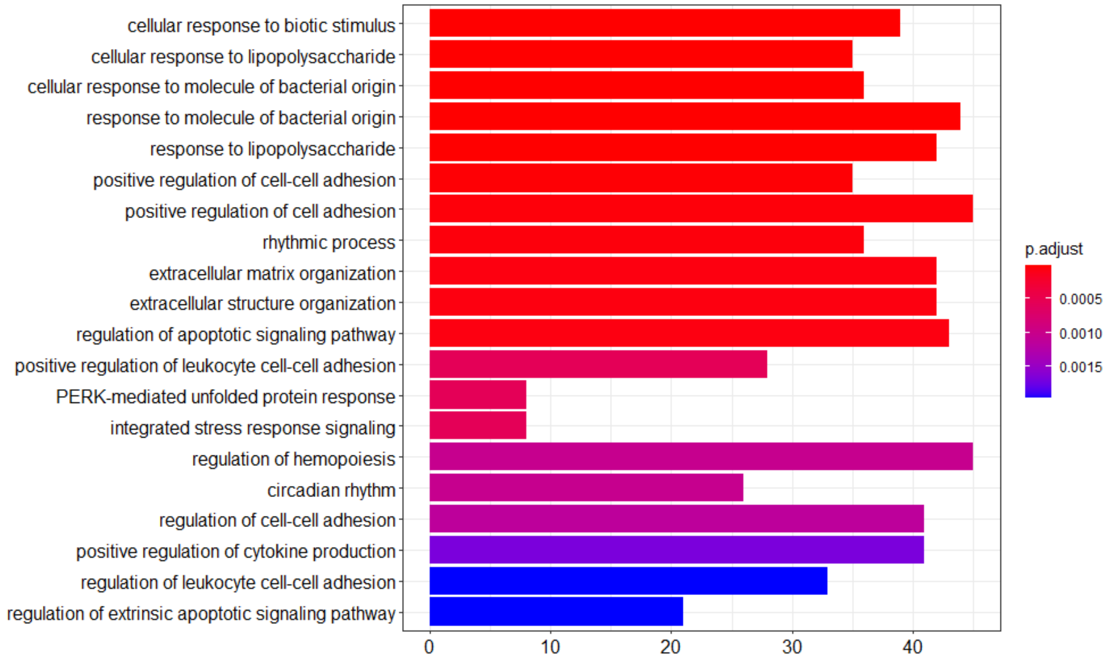

c

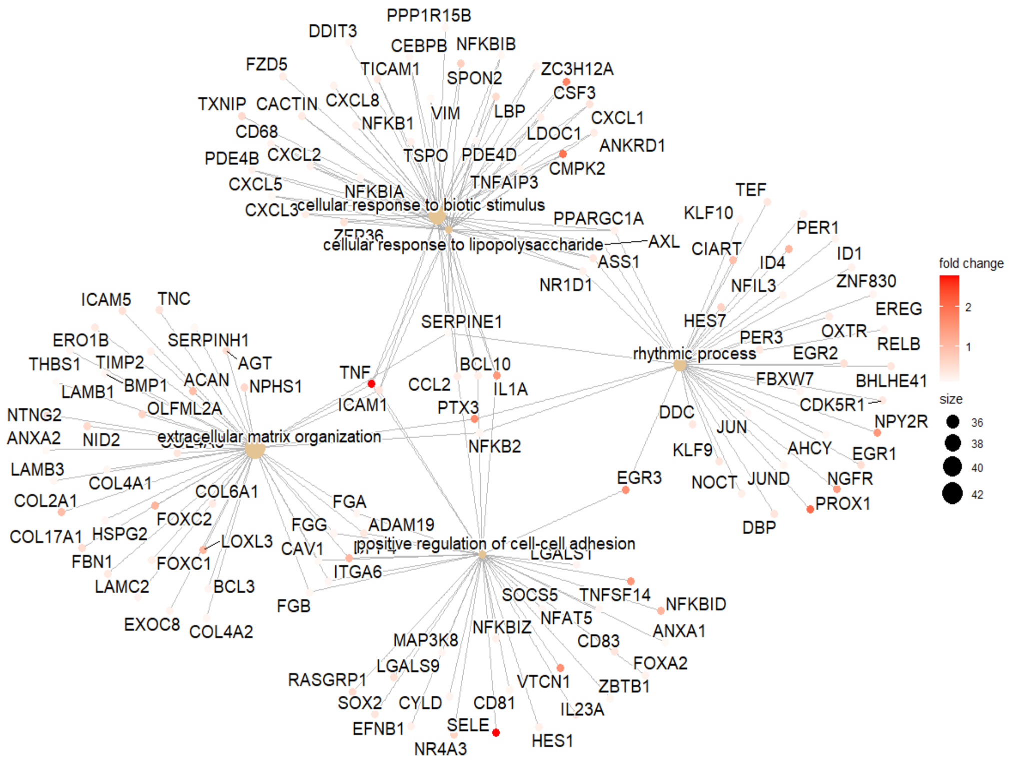

cellular response to biotic stimulus
cellular response to lipopolysaccharide
rhythmic process
extracellular matrix organization
positive regulation of cell-cell adhesion
PPP1R15B
DDIT3
CEBPB
NFKBIB
FZD5
TICAM1
SPON2
ZC3H12A
CSF3
TXNIP
CACTIN
CXCL8
VIM
LBP
CXCL1
CD68
NFKB1
LDOC1
ANKRD1
PDE4B
CXCL2
TSPO
PDE4D
CMPK2
CXCL5
NFKBIA
TNFAIP3
CXCL3
PPARGC1A
KLF10
TEF
AXL
PER1
CIART
ID1
ASS1
ID4
NR1D1
NFIL3
ZNF830
EREG
HES7
OXTR
PER3
RELB
EGR2
SERPINE1
ICAM5
TNC
ERO1B
SERPINH1
THBS1
TIMP2
AGT
BCL10
TNF
LAMB1
BMP1
ACAN
NPHS1
CCL2
IL1A
FBXW7
BHLHE41
OLFML2A
ICAM1
CDK5R1
NTNG2
PTX3
DDC
NPY2R
ANXA2
NID2
JUN
NFKB2
KLF9
AHCY
EGR1
LAMB3
COL4A1
EGR3
NOCT
JUND
NGFR
COL6A1
FGA
COL2A1
PROX1
FOXC2
ADAM19
FGG
DBP
COL17A1
HSPG2
LOXL3
CAV1
LGALS1
FBN1
FOXC1
ITGA6
LAMC2
BCL3
SOCS5
TNFSF14
NFKBID
FGB
NFAT5
EXOC8
COL4A2
NFKBIZ
ANXA1
MAP3K8
CD83
LGALS9
FOXA2
RASGRP1
VTCN1
ZBTB1
SOX2
CYLD
CD81
IL23A
EFNB1
SELE
HES1
NR4A3
fold change
2
1
size
36
38
40
42

d

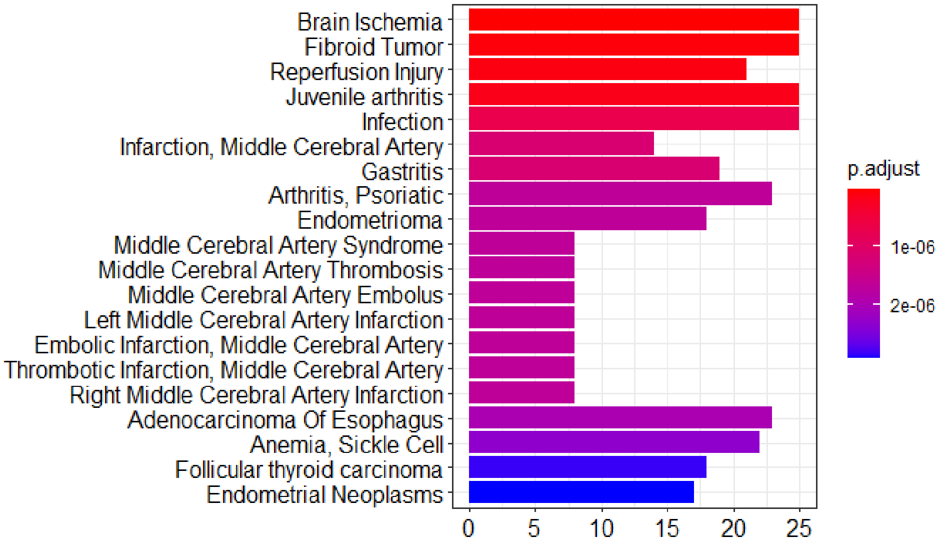

Brain Ischemia
Fibroid Tumor
Reperfusion Injury
Juvenile arthritis
Infection
Infarction, Middle Cerebral Artery
Gastritis
Arthritis, Psoriatic
Endometrioma
Middle Cerebral Artery Syndrome
Middle Cerebral Artery Thrombosis
Middle Cerebral Artery Embolus
Left Middle Cerebral Artery Infarction
Embolic Infarction, Middle Cerebral Artery
Thrombotic Infarction, Middle Cerebral Artery
Right Middle Cerebral Artery Infarction
Adenocarcinoma Of Esophagus
Anemia, Sickle Cell
Follicular thyroid carcinoma
Endometrial Neoplasms
0
5
10
15
20
25
p.adjust
1e-06
2e-06

Figure S4. Venn Diagram analysis of common genes in A549_A, Calu3_B, Calu3_C, and hCM (EGRs are in red).

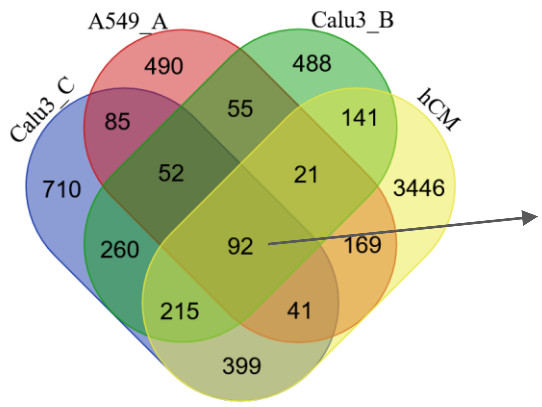

CXCL3 CMPK2 FRS2 USP42 IRF7 UPK1B NFAT5 SOCS3 TNFAIP3 GADD45B PPP1R15A CXCL2 CHD2 NR4A1 ZBTB10 PER1 FOSB TLE4 NFKBIA MAFF NR1D1 IRF1 GPCPD1 NEAT1 **EGR1** TM7SF2 ZC3H12A MAP3K8 **EGR2** HOGA1 PTX3 ACHE **EGR3** NOCT CCNL1 IFI6 FOS CXCL1 NFKBIE RSRC2 ARID4B JUN PPP4R4 ICAM5 GDF15 CDC14A INTS6 NFKB2 TNF IL1A NFKBIZ PTGS2 PIM3 ZC3H12C PRDM1 HELB PMAIP1 RELB MXD1 NFKB1 ATF3 CSF1 CXCL8 HDAC9 CCL20 CCL2 ARRDC3 JUNB ICAM1 ZEB2 CYLD SERPINE1 REL CREBRF BCL3

Figure S5. Metascape analysis of genes of interest. **a**. Common 10 genes to low viral load samples (Calu3_C, A549, hAE, and hBO) and their presence in Metascape database. **b**. The disease processes that are participated by the 10 genes. **c**. The above 10 genes and the 8 genes that common to samples of HEK293T_A, A549, A549_A, and Calu3_C and their presence in Metascape database. **d**. The presence of the 18 genes in all in Metascape database samples or patients' samples.

a

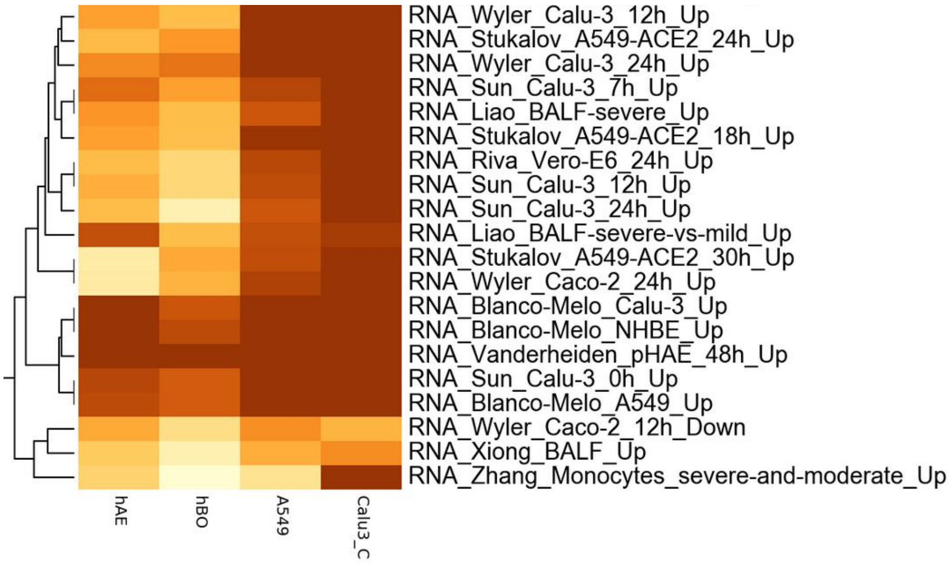

b

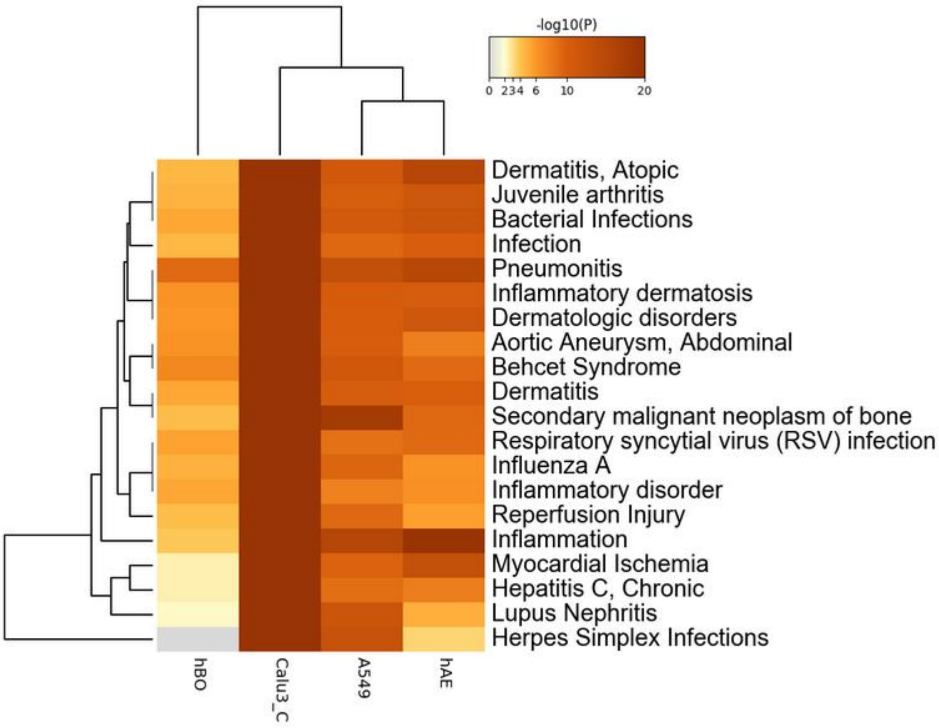

c

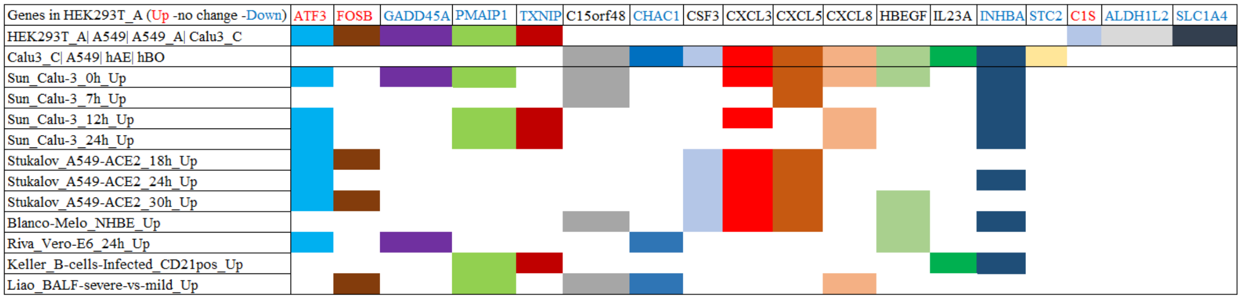

d

Coronascape datasets		TXNIP	EGR1	C1S	ATF3	PMAIP1	FOSB	INHBA	CSF3	GADD45A	STC2	CHAC1
All	362	43	19	17	15	13	12	10	8	5	5	4
Patients	131	30	4	1	0	2	6	0	2	0	0	1

Figure S6. GO and network analysis of DEGs of SARS-CoV-2 infection of A549 cells. **a.** Volcano plot of DEGs. **b.** Network view of the involvement of DEGs in the top 5 GO-BP categories. **c.** Upset plot of top 10 GO-BP**. d.** Dot plot of top 10 GO-BP, GO-CC, and GO-MF.

a

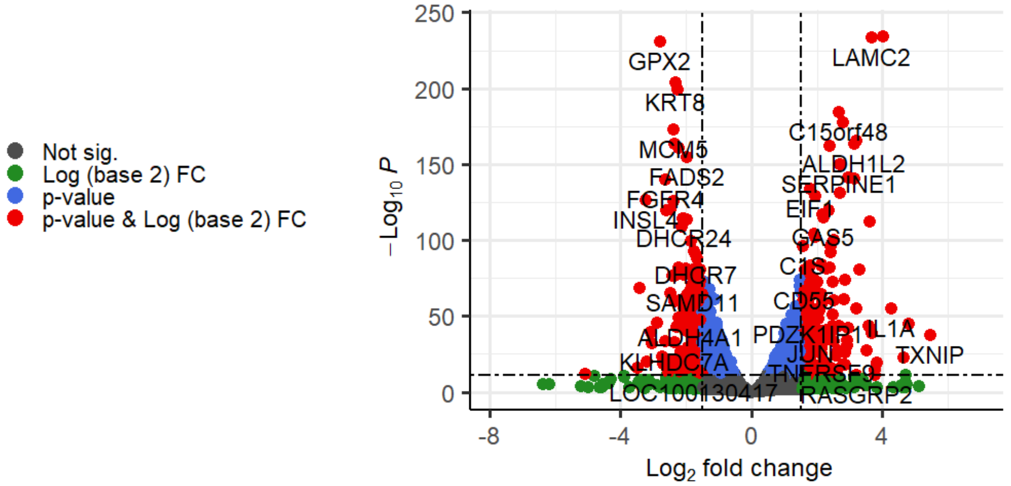

b

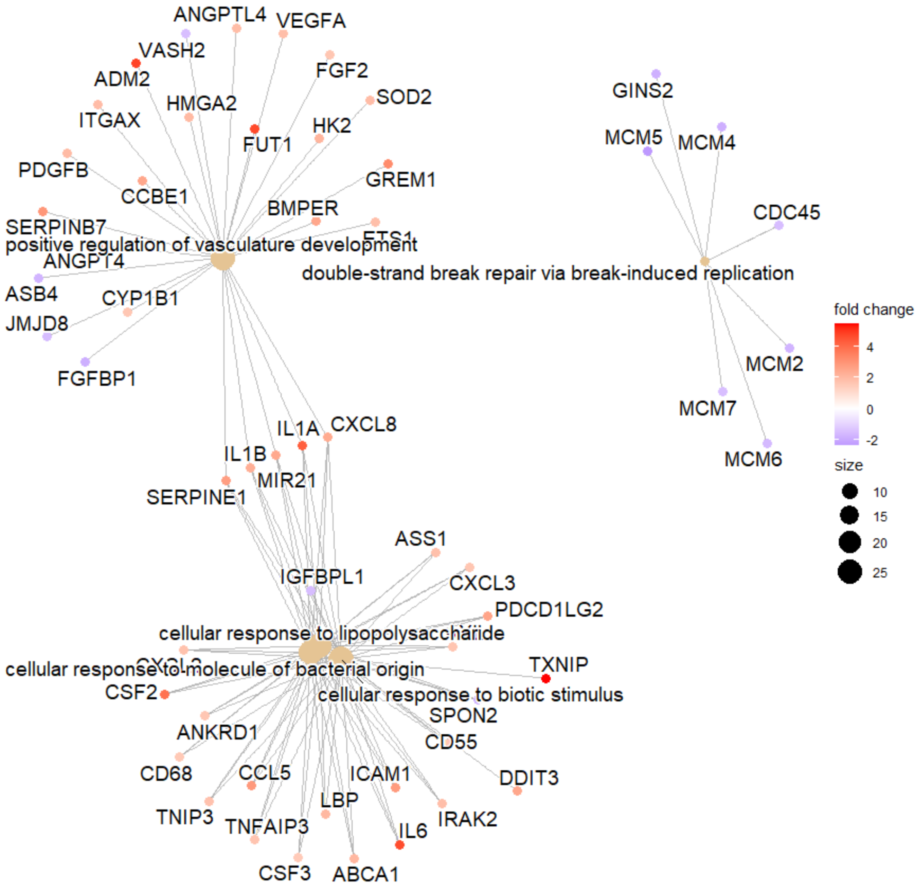

c

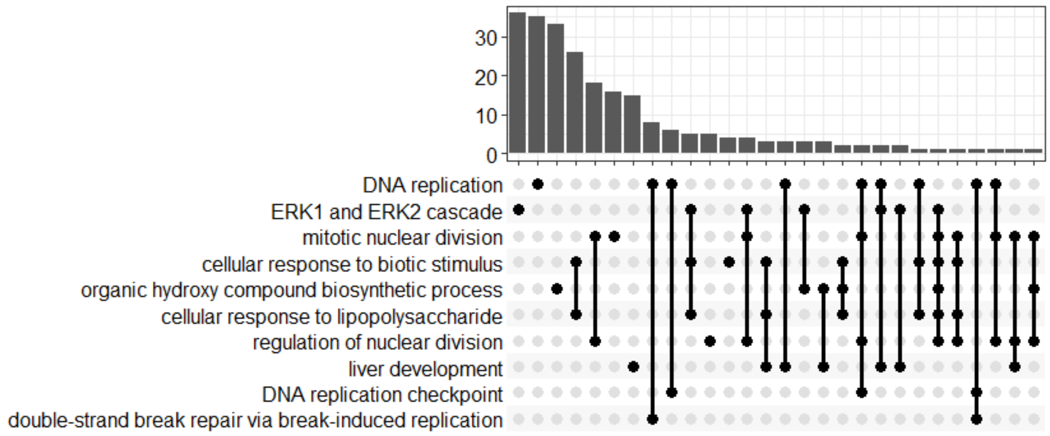

30
20
10
0
DNA replication
ERK1 and ERK2 cascade
mitotic nuclear division
cellular response to biotic stimulus
organic hydroxy compound biosynthetic process
cellular response to lipopolysaccharide
regulation of nuclear division
liver development
DNA replication checkpoint
double-strand break repair via break-induced replication

d

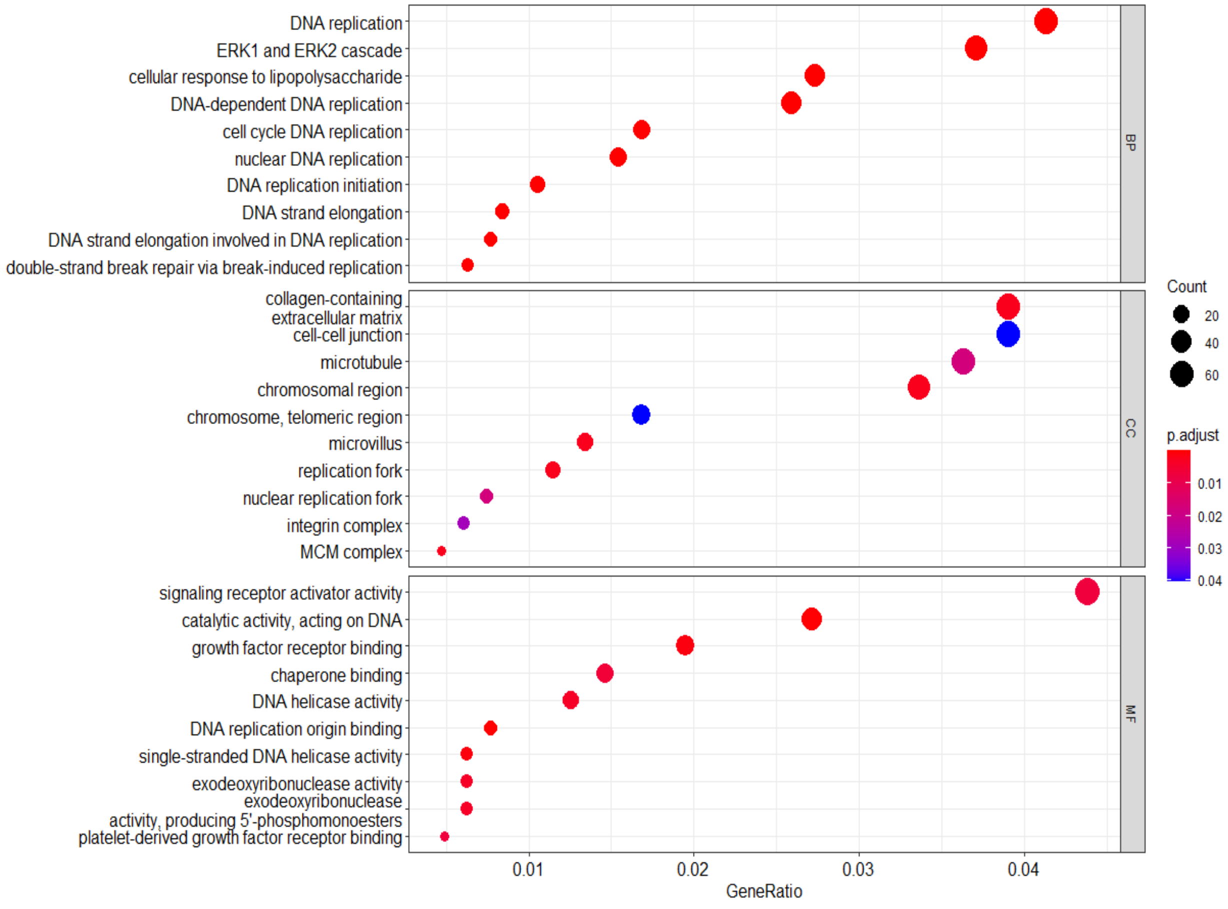

DNA replication
ERK1 and ERK2 cascade
cellular response to lipopolysaccharide
DNA-dependent DNA replication
cell cycle DNA replication
nuclear DNA replication
DNA replication initiation
DNA strand elongation
DNA strand elongation involved in DNA replication
double-strand break repair via break-induced replication
BP
collagen-containing extracellular matrix
cell-cell junction
microtubule
chromosomal region
chromosome, telomeric region
microvillus
replication fork
nuclear replication fork
integrin complex
MCM complex
CC
signaling receptor activator activity
catalytic activity, acting on DNA
growth factor receptor binding
chaperone binding
DNA helicase activity
DNA replication origin binding
single-stranded DNA helicase activity
exodeoxyribonuclease activity
exodeoxyribonuclease activity, producing 5'-phosphomonoesters
platelet-derived growth factor receptor binding
MF
0.01
0.02
0.03
0.04
GeneRatio
Count
20
40
60
p.adjust
0.01
0.02
0.03
0.04

Figure S7. GO and network analysis of DEGs of SARS-CoV-2 infection of Calu3_C cells. **a.** Volcano plot of DEGs. **b.** Upset plot of top 10 GO-BP**.** **c.** Dot plot of top 20 GO-BP. **d.** Network view of the involvement of DEGs in the top 5 GO-BP categories. **e.** Bar plot of DEGs in top 20 GO-CC. **f.** Bar plot plot of top 20 GO-MF**.**

a

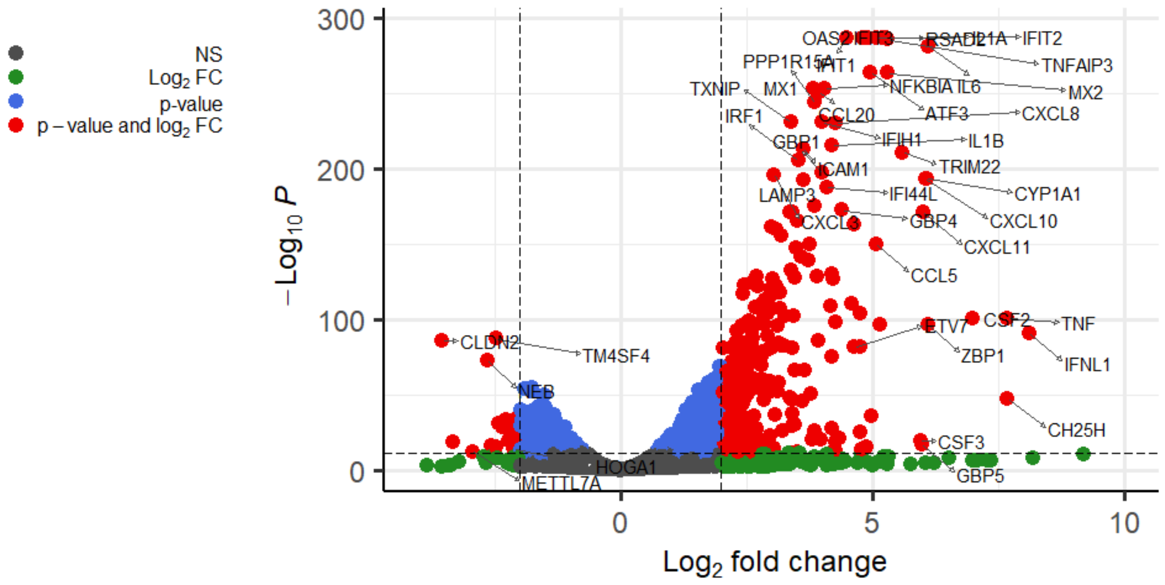

b

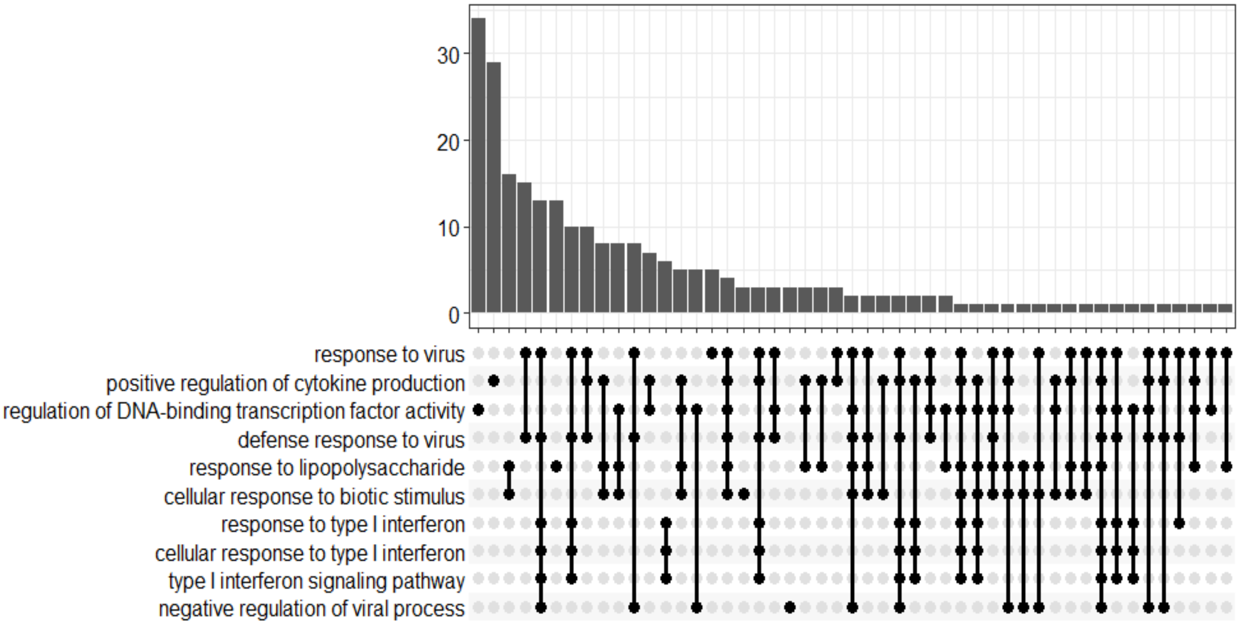

c

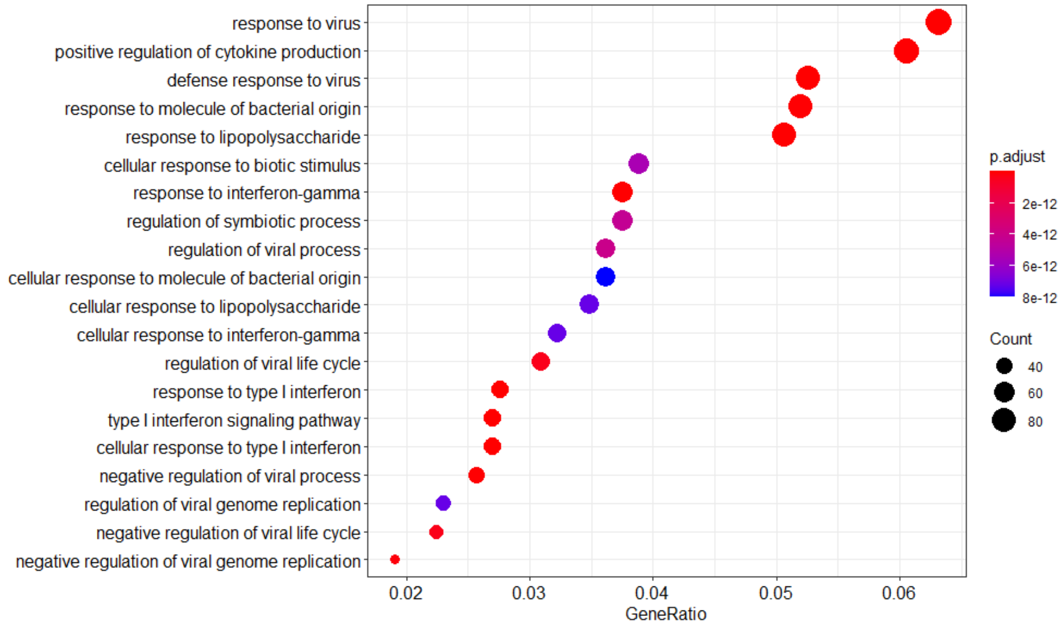

response to virus
positive regulation of cytokine production
defense response to virus
response to molecule of bacterial origin
response to lipopolysaccharide
cellular response to biotic stimulus
response to interferon-gamma
regulation of symbiotic process
regulation of viral process
cellular response to molecule of bacterial origin
cellular response to lipopolysaccharide
cellular response to interferon-gamma
regulation of viral life cycle
response to type I interferon
type I interferon signaling pathway
cellular response to type I interferon
negative regulation of viral process
regulation of viral genome replication
negative regulation of viral life cycle
negative regulation of viral genome replication
0.02
0.03
0.04
0.05
0.06
GeneRatio
p.adjust
2e-12
4e-12
6e-12
8e-12
Count
40
60
80

d

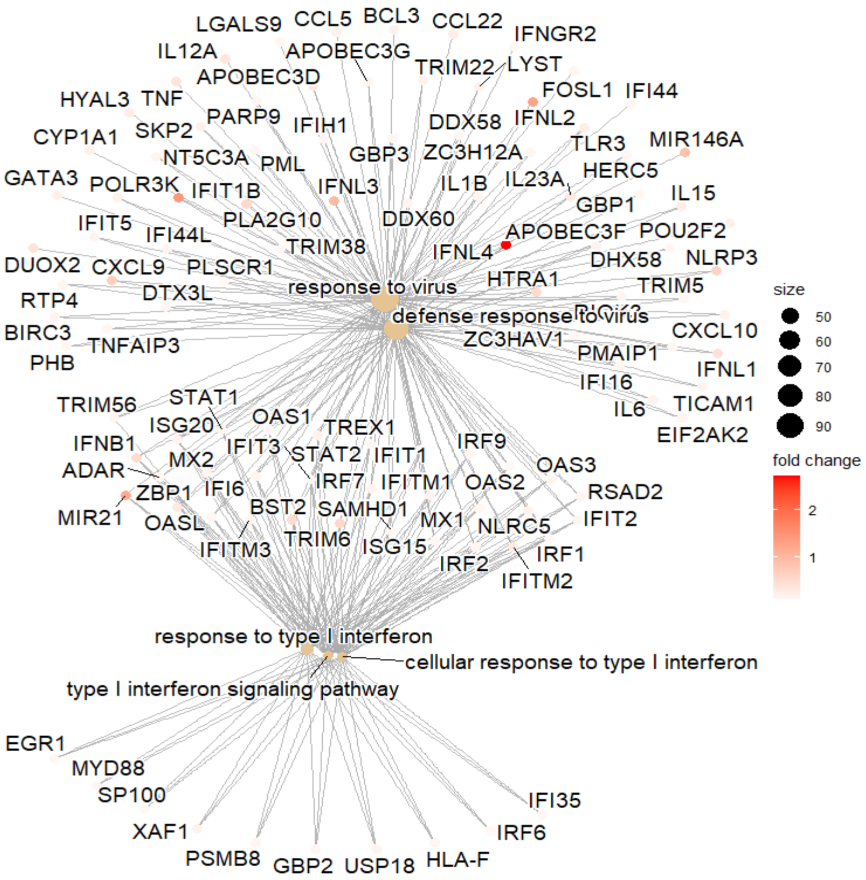

LGALS9
CCL5
BCL3
CCL22
IFNGR2
IL12A
APOBEC3G
TRIM22
LYST
APOBEC3D
FOSL1
IFI44
HYAL3
TNF
PARP9
IFIH1
DDX58
IFNL2
CYP1A1
SKP2
GBP3
ZC3H12A
TLR3
MIR146A
NT5C3A
PML
HERC5
GATA3
POLR3K
IFIT1B
IFNL3
IL1B
IL23A
GBP1
IL15
IFIT5
PLA2G10
DDX60
APOBEC3F
POU2F2
IFI44L
TRIM38
IFNL4
DHX58
NLRP3
DUOX2
CXCL9
PLSCR1
HTRA1
TRIM5
RTP4
DTX3L
response to virus
defense response to virus
BIRC3
ZC3HAV1
CXCL10
TNFAIP3
PMAIP1
PHB
IFNL1
IFI16
TRIM56
STAT1
IL6
TICAM1
ISG20
OAS1
TREX1
EIF2AK2
IFNB1
IFIT3
IFIT1
IRF9
MX2
STAT2
OAS3
ADAR
IRF7
IFITM1
OAS2
ZBP1
IFI6
RSAD2
MIR21
BST2
SAMHD1
OASL
MX1
NLRC5
IFIT2
IFITM3
TRIM6
ISG15
IRF1
IRF2
IFITM2
response to type I interferon
cellular response to type I interferon
type I interferon signaling pathway
EGR1
MYD88
SP100
IFI35
XAF1
IRF6
PSMB8
GBP2
USP18
HLA-F
size
50
60
70
80
90
fold change
2
1

e

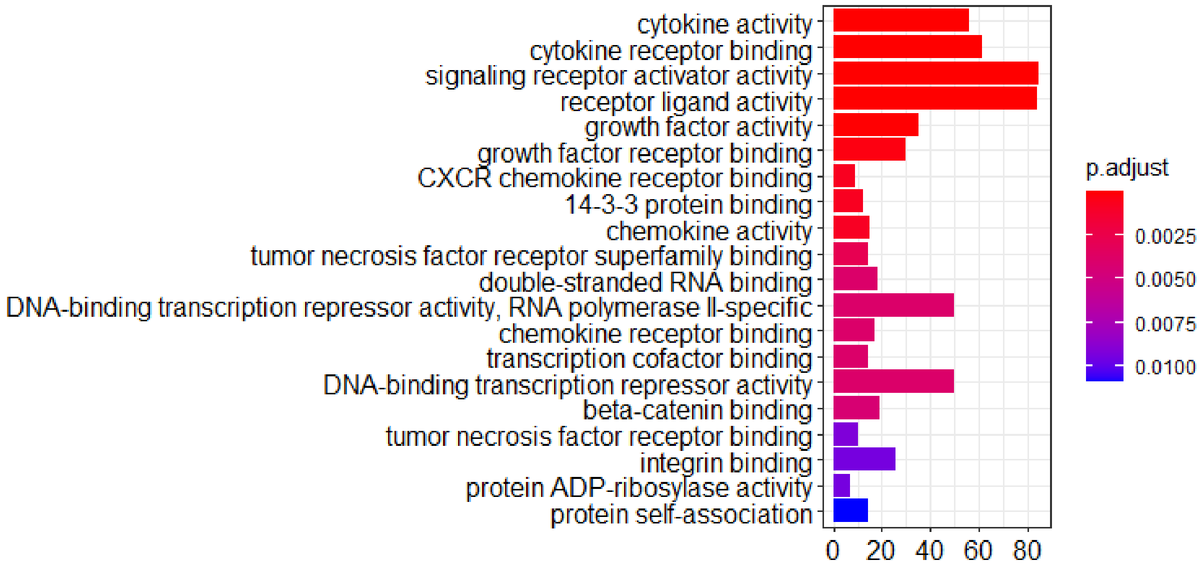
cytokine activity
cytokine receptor binding
signaling receptor activator activity
receptor ligand activity
growth factor activity
growth factor receptor binding
CXCR chemokine receptor binding
14-3-3 protein binding
chemokine activity
tumor necrosis factor receptor superfamily binding
double-stranded RNA binding
DNA-binding transcription repressor activity, RNA polymerase II-specific
chemokine receptor binding
transcription cofactor binding
DNA-binding transcription repressor activity
beta-catenin binding
tumor necrosis factor receptor binding
integrin binding
protein ADP-ribosylase activity
protein self-association
0 20 40 60 80
p.adjust
0.0025
0.0050
0.0075
0.0100

f

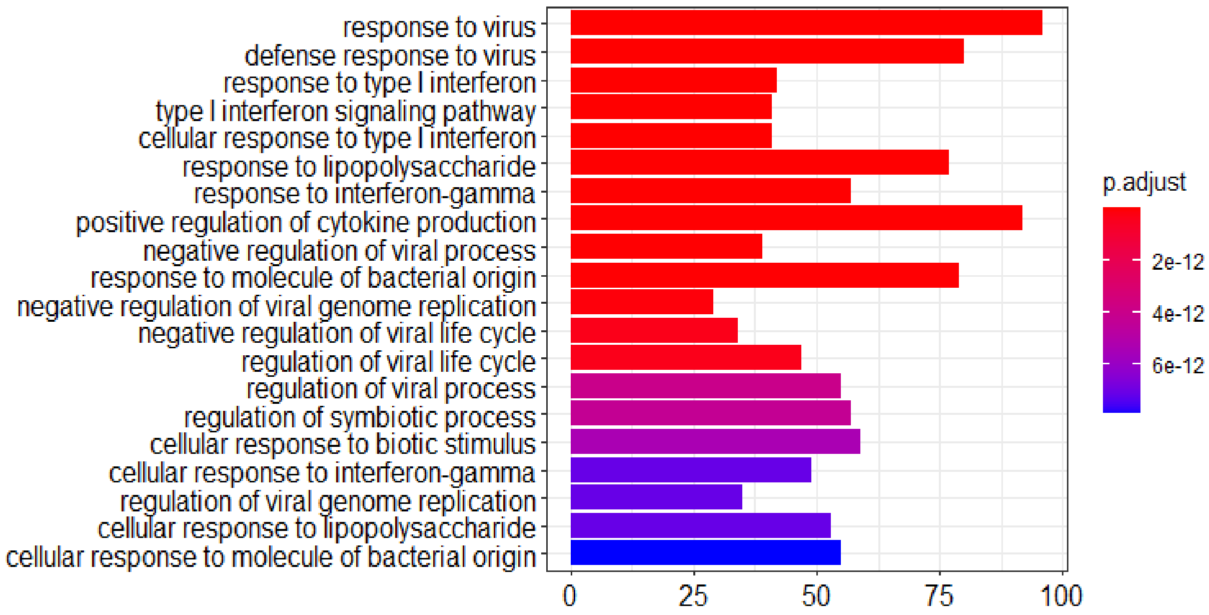
response to virus
defense response to virus
response to type I interferon
type I interferon signaling pathway
cellular response to type I interferon
response to lipopolysaccharide
response to interferon-gamma
positive regulation of cytokine production
negative regulation of viral process
response to molecule of bacterial origin
negative regulation of viral genome replication
negative regulation of viral life cycle
regulation of viral life cycle
regulation of viral process
regulation of symbiotic process
cellular response to biotic stimulus
cellular response to interferon-gamma
regulation of viral genome replication
cellular response to lipopolysaccharide
cellular response to molecule of bacterial origin
0 25 50 75 100
p.adjust
2e-12
4e-12
6e-12

Figure S8. GO and network analysis of DEGs of SARS-CoV-2 infection of hCM cells. **a.** Volcano plot of DEGs. **b.** Upset plot of top 10 GO-BP**. c.** Network view of the involvement of DEGs in the top 5 GO-BP categories. **d.** Dot plot of top 10 GO-BP, GO-CC, and GO-MF.

a

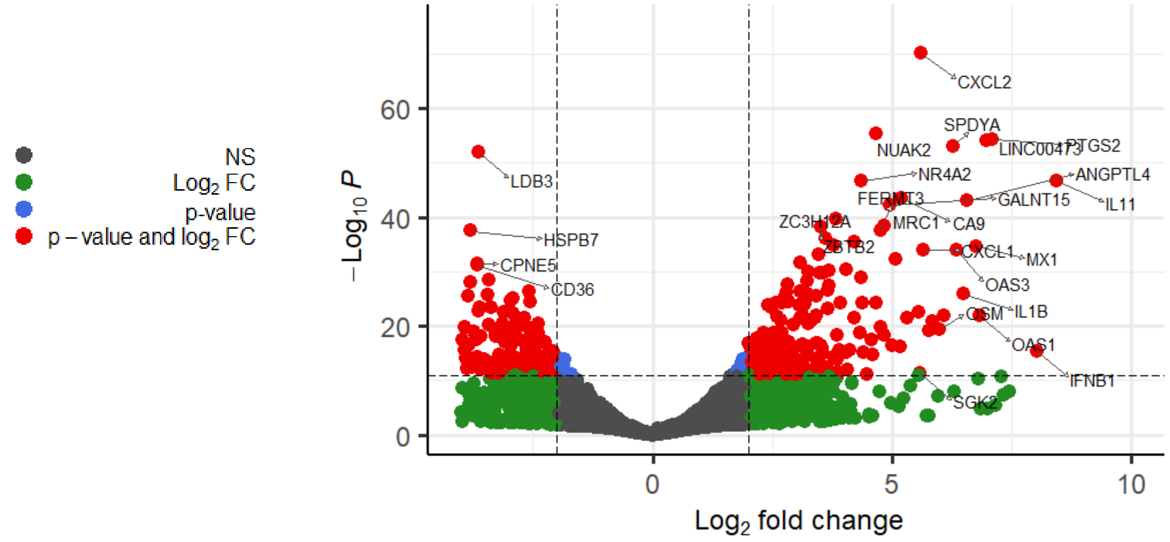

b

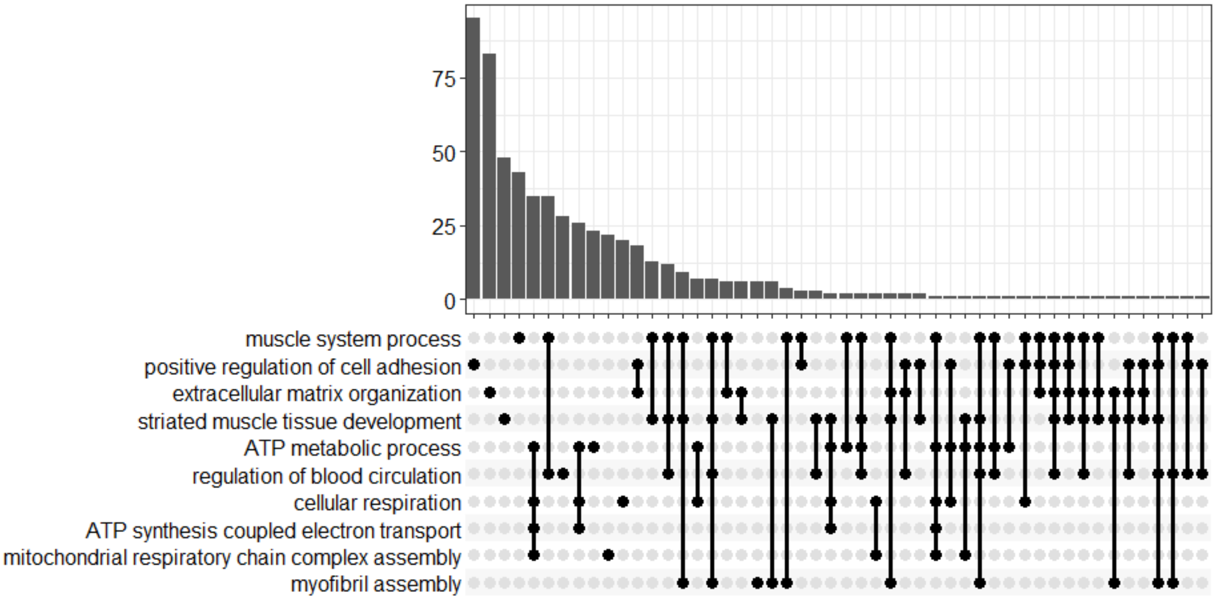

c

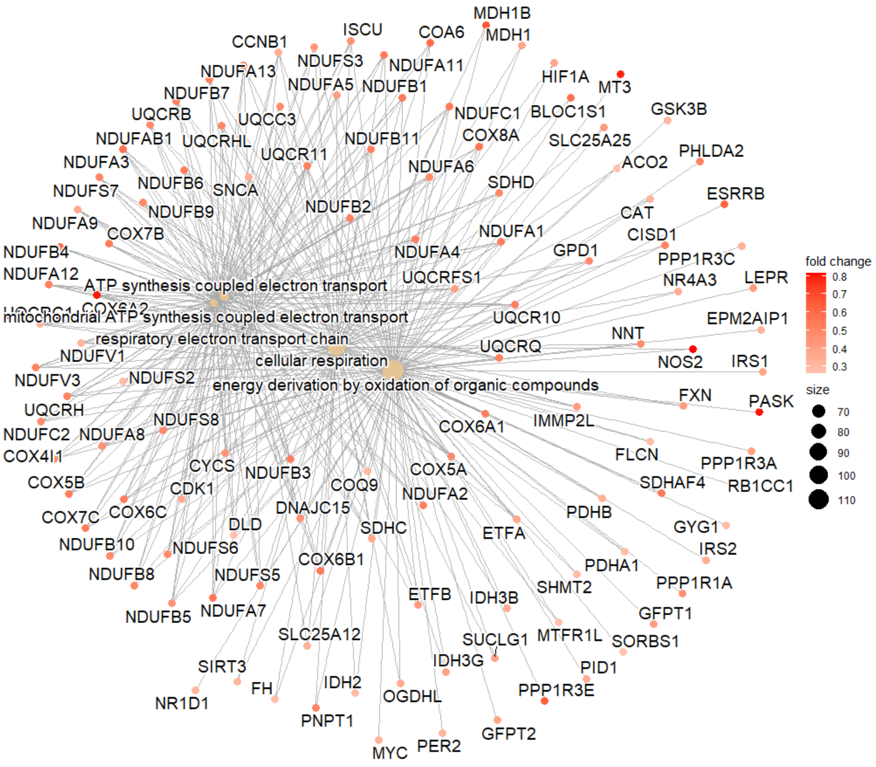
ATP synthesis coupled electron transport
mitochondrial ATP synthesis coupled electron transport
respiratory electron transport chain
cellular respiration
energy derivation by oxidation of organic compounds
MDH1B
MDH1
ISCU
COA6
CCNB1
NDUFS3
NDUFA11
NDUFA13
NDUFA5
NDUFB1
HIF1A
MT3
NDUFB7
UQCRB
UQCC3
NDUFC1
BLOC1S1
GSK3B
NDUFAB1
UQCRHL
NDUFB11
COX8A
SLC25A25
NDUFA3
UQCR11
NDUFA6
ACO2
PHLDA2
NDUFS7
NDUFB6
SNCA
SDHD
NDUFB9
ESRRB
NDUFA9
COX7B
NDUFB2
CAT
NDUFA1
CISD1
NDUFB4
NDUFA4
GPD1
PPP1R3C
NDUFA12
UQCRFS1
NR4A3
LEPR
UQCR10
EPM2AIP1
NDUFV1
UQCRQ
NNT
NOS2
IRS1
NDUFV3
NDUFS2
FXN
PASK
UQCRH
COX6A1
IMMP2L
NDUFC2
NDUFA8
NDUFS8
FLCN
PPP1R3A
COX4I1
CYCS
NDUFB3
COX5A
SDHAF4
RB1CC1
COX5B
CDK1
COQ9
NDUFA2
PDHB
COX7C
COX6C
DNAJC15
GYG1
DLD
SDHC
ETFA
IRS2
NDUFB10
NDUFS6
COX6B1
PDHA1
NDUFB8
NDUFS5
SHMT2
PPP1R1A
NDUFB5
NDUFA7
ETFB
IDH3B
GFPT1
SLC25A12
SUCLG1
MTFR1L
SORBS1
SIRT3
IDH3G
PID1
NR1D1
FH
IDH2
OGDHL
PPP1R3E
PNPT1
GFPT2
MYC
PER2
fold change
0.8
0.7
0.6
0.5
0.4
0.3
size
70
80
90
100
110

d

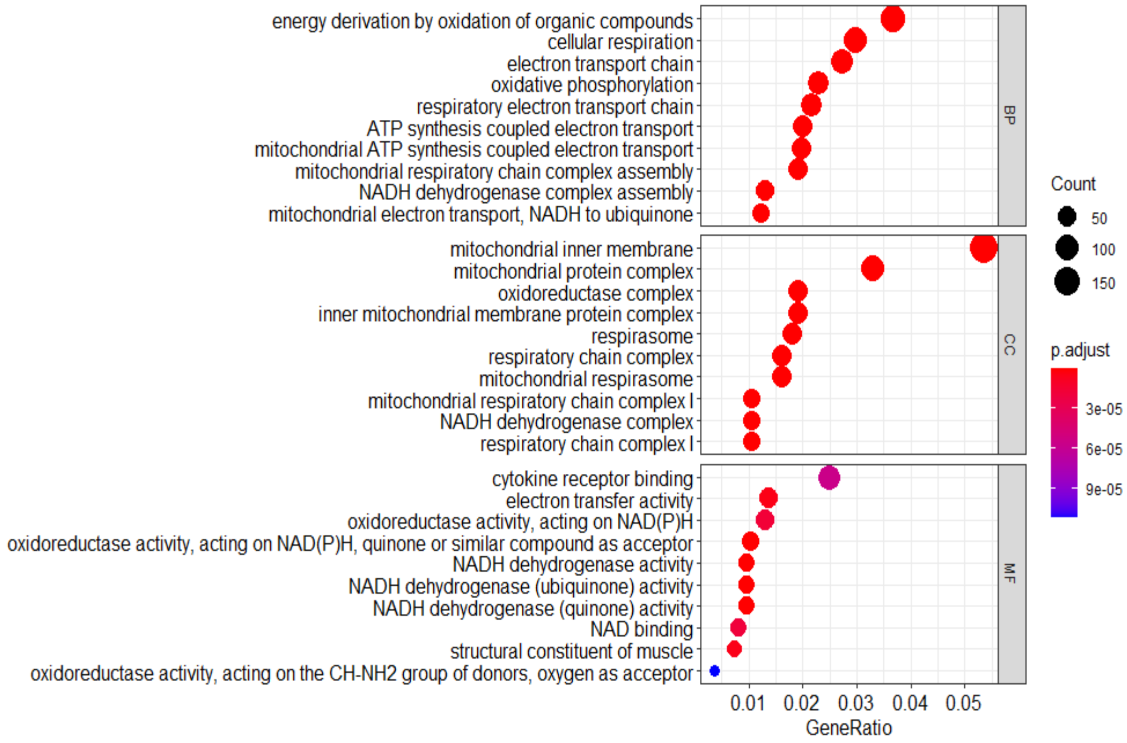
energy derivation by oxidation of organic compounds
cellular respiration
electron transport chain
oxidative phosphorylation
respiratory electron transport chain
ATP synthesis coupled electron transport
mitochondrial ATP synthesis coupled electron transport
mitochondrial respiratory chain complex assembly
NADH dehydrogenase complex assembly
mitochondrial electron transport, NADH to ubiquinone
mitochondrial inner membrane
mitochondrial protein complex
oxidoreductase complex
inner mitochondrial membrane protein complex
respirasome
respiratory chain complex
mitochondrial respirasome
mitochondrial respiratory chain complex I
NADH dehydrogenase complex
respiratory chain complex I
cytokine receptor binding
electron transfer activity
oxidoreductase activity, acting on NAD(P)H
oxidoreductase activity, acting on NAD(P)H, quinone or similar compound as acceptor
NADH dehydrogenase activity
NADH dehydrogenase (ubiquinone) activity
NADH dehydrogenase (quinone) activity
NAD binding
structural constituent of muscle
oxidoreductase activity, acting on the CH-NH2 group of donors, oxygen as acceptor
BP
CC
MF
0.01
0.02
0.03
0.04
0.05
GeneRatio
Count
50
100
150
p.adjust
3e-05
6e-05
9e-05

Figure S9. GO and network analysis of DEGs of SARS-CoV-2 infection of hAE cells. **a.** Volcano plot of DEGs. **b.** Upset plot of top 10 GO-BP**. c.** Network view of the involvement of DEGs in the top 5 GO-BP categories. **d.** Dot plot of top 10 GO-BP, GO-CC, and GO-MF.

a

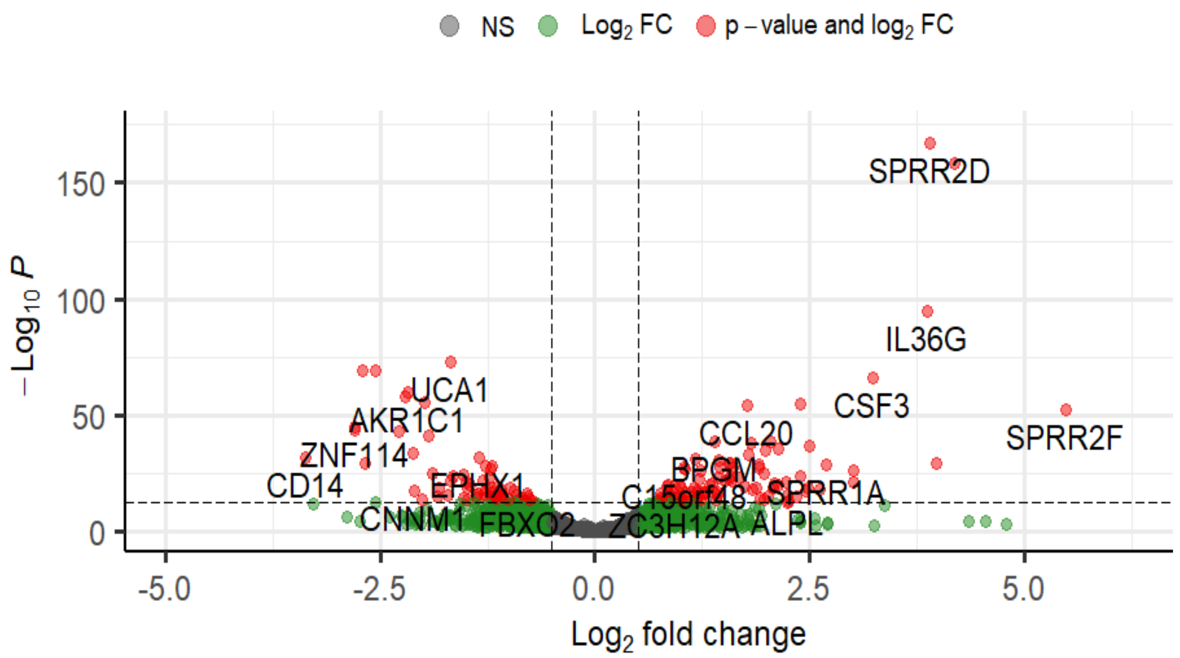

b

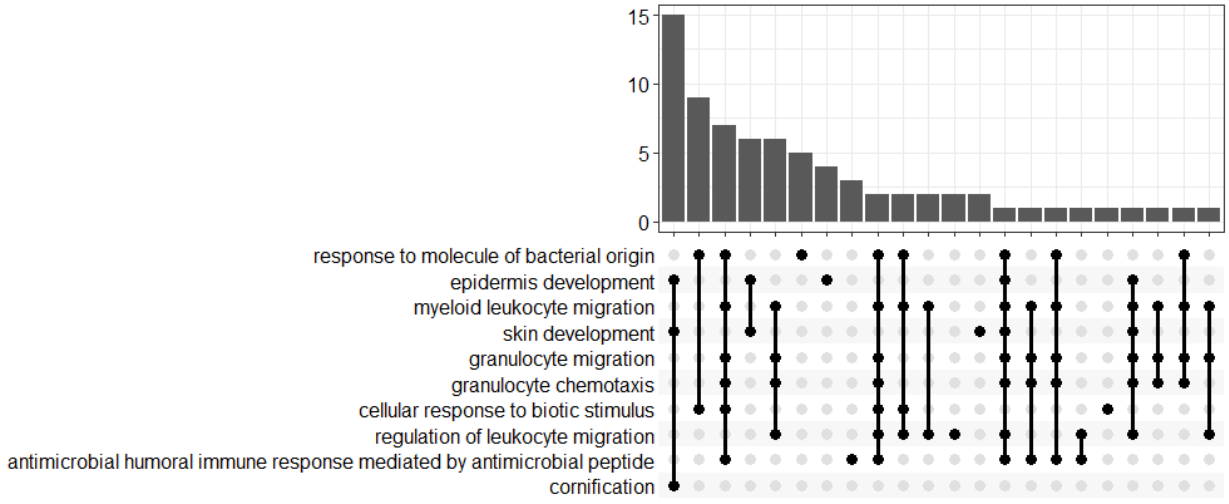

c

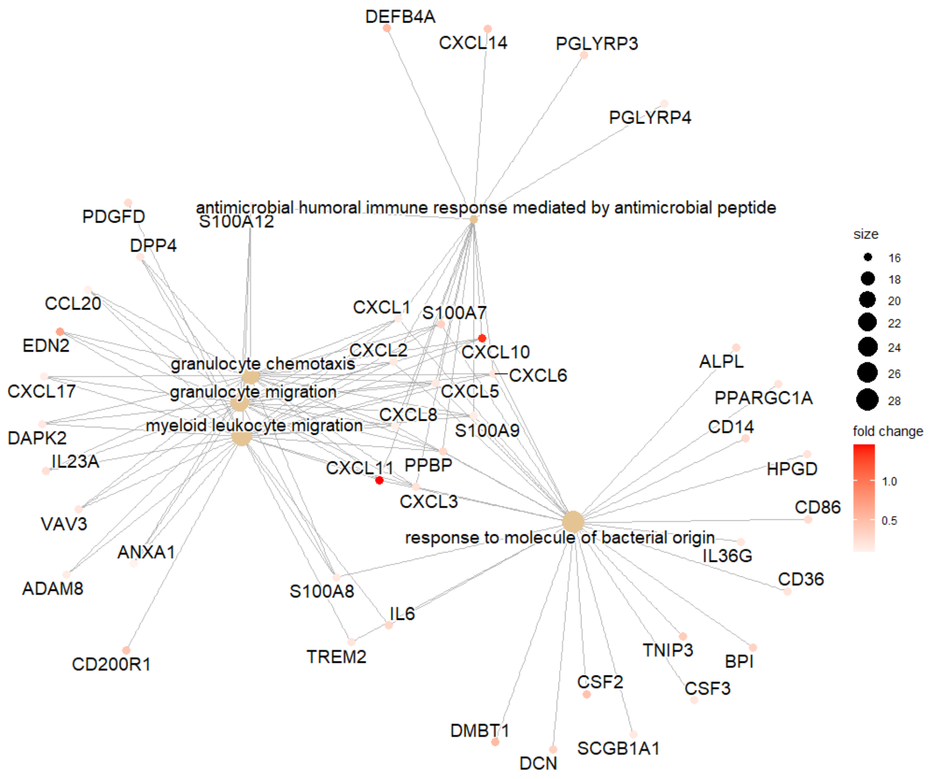

DEFB4A
CXCL14
PGLYRP3
PGLYRP4
antimicrobial humoral immune response mediated by antimicrobial peptide
S100A12
PDGFD
DPP4
CCL20
EDN2
CXCL17
DAPK2
IL23A
VAV3
ANXA1
ADAM8
CD200R1
CXCL1
S100A7
CXCL2
CXCL10
CXCL6
granulocyte chemotaxis
granulocyte migration
myeloid leukocyte migration
CXCL5
CXCL8
S100A9
CXCL11
PPBP
CXCL3
ALPL
PPARGC1A
CD14
HPGD
CD86
response to molecule of bacterial origin
IL36G
CD36
S100A8
IL6
TREM2
TNIP3
BPI
CSF2
CSF3
DMBT1
SCGB1A1
DCN
size
16
18
20
22
24
26
28
fold change
1.0
0.5

d

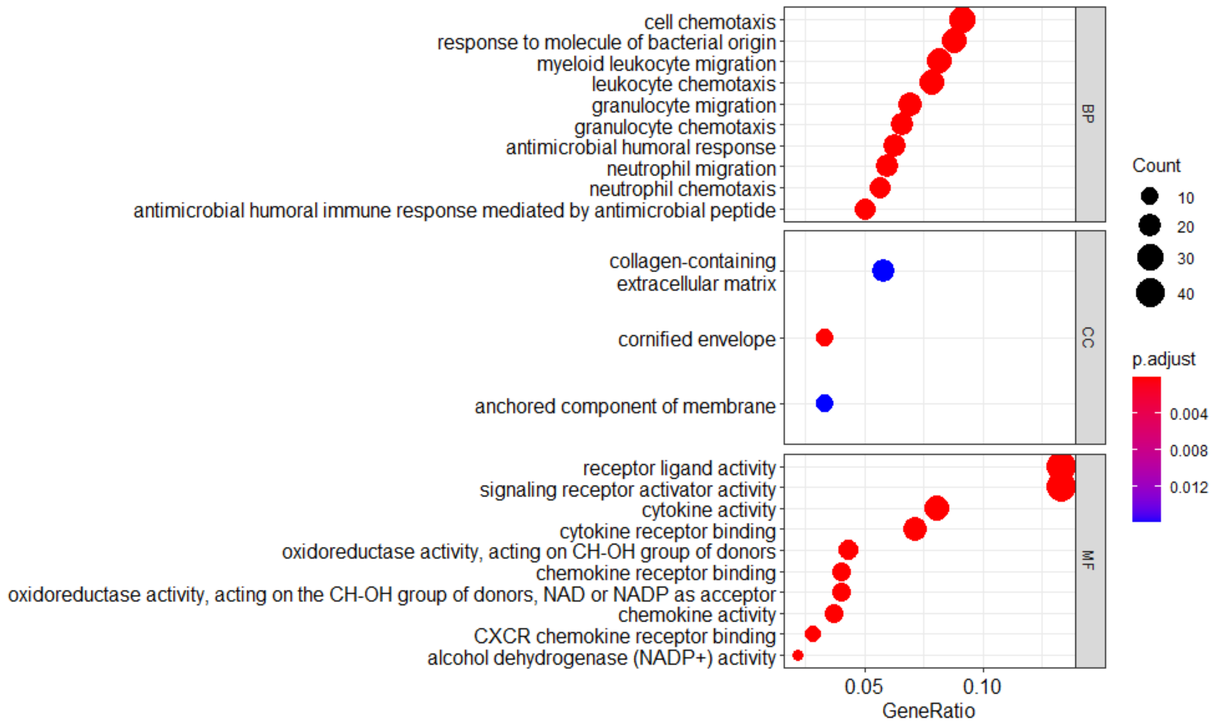

cell chemotaxis
response to molecule of bacterial origin
myeloid leukocyte migration
leukocyte chemotaxis
granulocyte migration
granulocyte chemotaxis
antimicrobial humoral response
neutrophil migration
neutrophil chemotaxis
antimicrobial humoral immune response mediated by antimicrobial peptide
collagen-containing extracellular matrix
cornified envelope
anchored component of membrane
receptor ligand activity
signaling receptor activator activity
cytokine activity
cytokine receptor binding
oxidoreductase activity, acting on CH-OH group of donors
chemokine receptor binding
oxidoreductase activity, acting on the CH-OH group of donors, NAD or NADP as acceptor
chemokine activity
CXCR chemokine receptor binding
alcohol dehydrogenase (NADP+) activity
BP
CC
MF
0.05
0.10
GeneRatio
Count
10
20
30
40
p.adjust
0.004
0.008
0.012

Figure S10. Comparison analyses of DEG in A549, A549-hACE2 (A549_A), Caco2, Calu3, hCM, and hBO cells. Heatmap view of genes with the abs(log2FC values) ≥ 2 in SARS-CoV-2 infected **a.** A549 cells, **b.** A549_A cells, **c.** Caco2 cells, **d.** Calu3_B cells, **e.** Calu3_C, and **f.** hCM cells versus these genes in other infected cell lines. Grey color indicates the log2FC values are not available in that cell lines. **g**. Circular plot of DEGs in SRAS-CoV-2 infection of A549, hBO, hAE and Calu3_C cells.

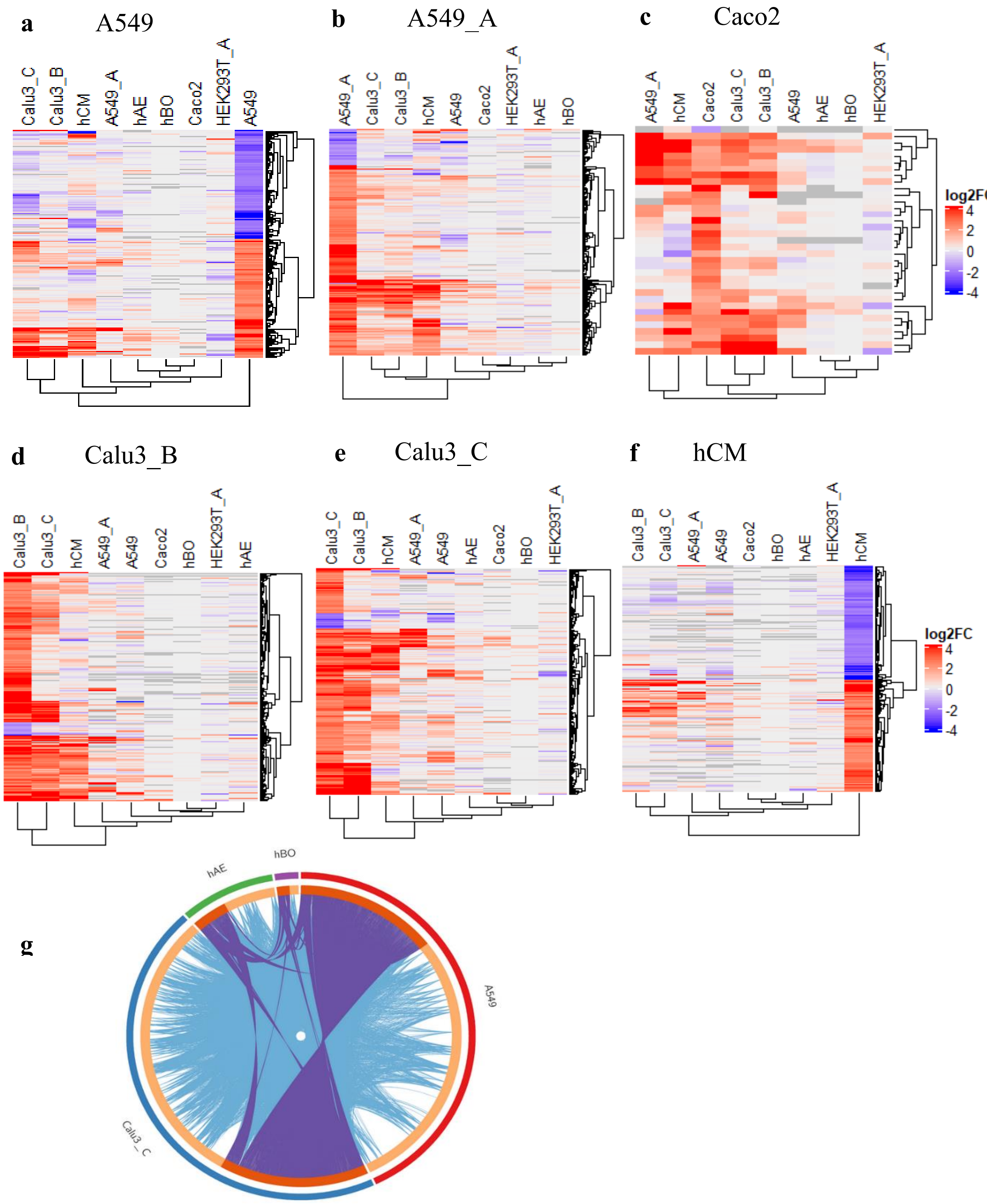

Figure S11. Venn Diagram analysis of common genes in Calu3_B, Calu3_C, and hCM

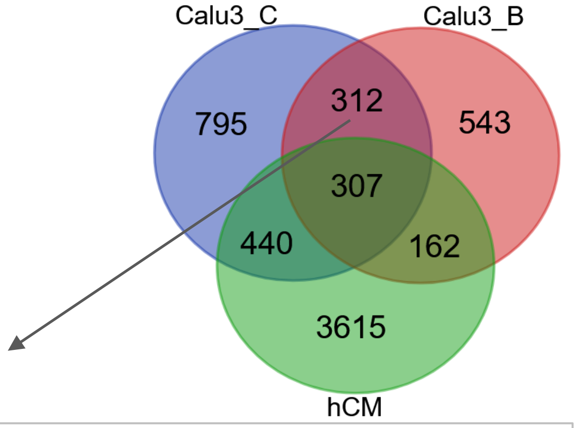

CLK4 CXCL3 PNPT1 CAPS NR4A2 CMPK2 EGOT GFPT2 TNFRSF9 FRS2 USP42 CYP1A1 ATP10A KLF4 OAS1 DLGAP1 IRF7 UPK1B HERC6 CASP1 XAF1 MYCL PLA2G4C SECTM1 NFAT5 IL15RA LOC100130476 SNCA LINC00881 CSRNP1 NR4A3 SOCS3 THEMIS2 AMOTL2 TNFAIP3 GBP4 CA12 GADD45B UCP2 EGR4 EIF2AK2 PPP1R15A BATF2 PTGER2 IRAK2 SP110 SAMHD1 CXCL2 RASGEF1B PRKD2 CHD2 NR4A1 NFE2L3 PALMD TTC39B OAS2 IPCEF1 CRIP2 HRH1 ZBTB10 PDZD2 ADAR SLC15A3 INHBE IRF2 CXCL10 HERC5 ANKRD33B PER1 FOSB FAM46C DDO PDCD1LG2 TLE4 NFKBIA GBP5 NCOA7 ZNF804A MT2A HSD17B14 MAFF TYMP NR1D1 TRIM5 CH25H IRF1 C8orf4 GPCPD1 STAT4 TAP1 ETS2 NEAT1 EGR1 RIPK2 SELE TM7SF2 ABTB2 ZC3H12A MAP3K8 EGR2 HOGA1 VEGFC SAMD9L HELZ2 DUSP1 TCTEX1D2 PTX3 UBD PELI1 ACHE EGR3 OASL ACTN2 IL6 ISG15 ITGAM C19orf66 NOCT BST2 CCNL1 IFI6 HIVEP2 SLC5A5 LOC541472 HAND1 IFI44L FOS KCNN1 TNFRSF1B TRIM38 ISG20 CXCL1 RTP4 IFI35 GBP3 DDX60L TNFAIP2 NFKBIE DDX58 RGMB-AS1 SHROOM2 RSRC2 EGLN3 SLFN5 LINC00880 SAMD9 ARID4B JUN TRIM25 GATM RND1 PPP4R4 ICAM5 IL7 BIRC3 GDF15 SP140L SCAMP1-AS1 CDC14A INTS6 BISPR IFNL1 SERPINB9 BTN3A1 NFKB2 SBNO2 C1orf168 IKZF3 IFI16 TNF IL1A NFKBIZ ETV7 PTGS2 IL4I1 USP18 LST1 IRS2 PIM3 MXRA5 EPSTI1 TRANK1 TRIM14 ZC3H12C STAT1 TNFRSF25 PTGER4 PRDM1 GATA3 PARP12 C8orf46 SUSD3 ARHGEF28 C6orf222 SOGA3 GBP1 HELB PMAIP1 GSDMB CCL5 MX1 RELB IL32 SPSB1 ASPHD2 MXD1 IFIH1 NUAK2 NFKB1 TDRD7 SLCO5A1 C11orf96 CEACAM1 NOD2 GPR68 DHX58 IFITM1 IL18R1 PARP9 BACH2 HAP1 PLAUR C1QTNF1 IFIT3 LOC286059 KMT2E TICAM1 SYNPO2 TRIM22 NLRP3 ATF3 APOL6 UBA7 CSF1 TNFSF10 DTX3L TBC1D22B ARID5B CXCL11 CXCL8 HDAC9 PARP14 IFIT2 ZC3HAV1 KSR1 OAS3 WARS LMO2 DDX60 CCL20 STX11 PCK1 CTH IL15 TRIM69 RND3 TRERF1 IL12A HEG1 CCL2 CHAC1 MX2 CREB5 SYT1 KCNT2 MYD88 PML FAP SMURF1 HDX INHBA LOC100506178 SP100 NLRC5 LIFR ARRDC3 IFIT1 JUNB TNFSF13B SPDEF PARP10 ICAM1 LGALS9 TRAF1 ZEB2 CYLD SERPINE1 VCAM1 STAT2 REL CREBRF RSAD2 NCALD NEDD9 NPTX1 BCL3 DUSP16 GPT2 SAA2 ZNFX1 IFNB1 GEM KCNJ15

3. Supplemental data file 1. HEK293T-hACE2_genes_count.xlsx contains raw gene counts from subread2 for HEK293T-hACE2.

4. Supplemental data file 2. DEseq2 results_condition_cov_vs_mock.xlsx contains DEseq2 produced differential gene expression (viral infection versus mock infection) results for all datasets.
